# Correction of osteopetrosis in the neonate *oc/oc* murine model after lentiviral vector gene therapy and non-genotoxic conditioning

**DOI:** 10.3389/fendo.2024.1450349

**Published:** 2024-09-09

**Authors:** Sara Penna, Alessandra Zecchillo, Martina Di Verniere, Elena Fontana, Valeria Iannello, Eleonora Palagano, Stefano Mantero, Andrea Cappelleri, Elena Rizzoli, Ludovica Santi, Laura Crisafulli, Marta Filibian, Antonella Forlino, Luca Basso-Ricci, Serena Scala, Eugenio Scanziani, Thorsten Schinke, Francesca Ficara, Cristina Sobacchi, Anna Villa, Valentina Capo

**Affiliations:** ^1^ San Raffaele Telethon Institute for Gene Therapy (SR-Tiget), IRCCS San Raffaele Scientific Institute, Milan, Italy; ^2^ Translational and Molecular Medicine (DIMET), University of Milano Bicocca, Milan, Italy; ^3^ Milan Unit, Istituto di Ricerca Genetica e Biomedica, Consiglio Nazionale delle Ricerche, Milan, Italy; ^4^ Humanitas Research Hospital IRCCS, Rozzano, MI, Italy; ^5^ Vita-Salute San Raffaele University, Milan, Italy; ^6^ Florence Unit, Istituto di Bioscienze e Biorisorse, Consiglio Nazionale delle Ricerche, Sesto Fiorentino, Italy; ^7^ Mouse and Animal Pathology Laboratory, UniMi Foundation, Milan, Italy; ^8^ Department of Veterinary Medicine and Animal Sciences, University of Milan, Lodi, Italy; ^9^ Biomedical Imaging Laboratory, Centro Grandi Strumenti, University of Pavia, Pavia, Italy; ^10^ Department of Molecular Medicine, Biochemistry Unit, University of Pavia, Pavia, Italy; ^11^ Department of Osteology and Biomechanics, University Medical Center Hamburg-Eppendorf, Hamburg, Germany

**Keywords:** gene therapy, osteopetrosis, lentiviral vector, osteoclast, hematopoietic stem cells, HSC mobilization, conditioning, *TCIRG1* gene

## Abstract

**Introduction:**

Autosomal recessive osteopetrosis (ARO) is a rare genetic disease, characterized by increased bone density due to defective osteoclast function. Most of the cases are due to *TCIRG1* gene mutation, leading to severe bone phenotype and death in the first years of life. The standard therapy is the hematopoietic stem cell transplantation (HSCT), but its success is limited by several constraints. Conversely, gene therapy (GT) could minimize the immune-mediated complications of allogeneic HSCT and offer a prompt treatment to these patients.

**Methods:**

The *Tcirg1*-defective *oc/oc* mouse model displays a short lifespan and high bone density, closely mirroring the human condition. In this work, we exploited the *oc/oc* neonate mice to optimize the critical steps for a successful therapy.

**Results:**

First, we showed that lentiviral vector GT can revert the osteopetrotic bone phenotype, allowing long-term survival and reducing extramedullary haematopoiesis. Then, we demonstrated that plerixafor-induced mobilization can further increase the high number of HSPCs circulating in peripheral blood, facilitating the collection of adequate numbers of cells for therapeutic purposes. Finally, pre-transplant non-genotoxic conditioning allowed the stable engraftment of HSPCs, albeit at lower level than conventional total body irradiation, and led to long-term survival and correction of bone phenotype, in the absence of acute toxicity.

**Conclusion:**

These results will pave the way to the implementation of an effective GT protocol, reducing the transplant-related complication risks in the very young and severely affected ARO patients.

## Introduction

Autosomal recessive osteopetrosis (ARO) is an inherited bone disorder that results from a defect in osteoclast (OC) differentiation or function, leading to increased bone density. Symptoms are severe and include dense and brittle bones with dramatic fibrosis of the bone marrow (BM) cavity, severe anemia, hepatosplenomegaly, macrocephaly, progressive deafness and blindness due to cranial nerves compression (1–3). The molecular bases of the disease are heterogenous but the majority of ARO patients carry mutations in the *TCIRG1* gene. This gene encodes the a3 subunit of the V0 complex of the ATPase proton pump, necessary for the acidification of the bone resorption lacunae and consequently for OC bone resorptive function (4, 5). The murine model of the disease is the *oc/oc* osteopetrotic mouse, which carries a 1.6-kb deletion at the 5’ end of the *TCIRG1* gene that abolishes the transcription of the ATPase proton pump subunit and leads to the absence of the enzyme on the apical membrane of OCs (6, 7). The mouse model closely resembles the human disease and is characterized by short life expectancy (less than 3 weeks), failure to thrive, absence of incisor eruption, clubbed feet, circling behavior, generalized increase of skeletal density with fibrosis of the BM cavities, extensive extramedullary hematopoiesis, hypocalcemia and hyperparathyroidism (6–8).

Currently hematopoietic stem cell transplantation (HSCT) is the only therapeutic option for ARO patients. Despite recent advances in conditioning protocols, transplant-related mortality remains high, because the success of the transplant depends on the donor compatibility, the age of the patient and the severity of pre-existing irreversible medical conditions (9–12). Being the timing of the transplant of fundamental importance, gene therapy (GT) has been proposed as an alternative option for ARO patient. Clinically-optimized *TCIRG1*-expressing lentiviral vectors (LVs) have been used to transduce the hematopoietic stem and progenitor cells (HSPCs) of ARO patients and proved able to restore the function of differentiated osteoclasts *in vitro* (13, 14). *In vivo* efficacy of GT, in terms of improved survival and bone phenotype, has been shown in *oc/oc* mice treated with a lentiviral vector expressing the human *TCIRG1* under the control of the elongation factor 1α short (EFS) promoter (15). Based on these results, a phase I clinical trial (NCT04525352) opened in 2020, but was then discontinued after the early death of the first treated patient for gene therapy unrelated causes (16).

In ARO condition, bone marrow (BM) harvest is precluded by the dense bone sclerosis of BM cavity and bone fragility. CD34+ cells from mobilized peripheral blood represent the sole HSPC source available for *ex vivo* manipulation. Notably, ARO patients usually present a high frequency of circulating CD34+ cells in their blood (13, 17, 18), that may be sufficient, even without drug-induced mobilization, for GT protocols if coupled to HSPC cell expansion (13). Up to date, ARO spontaneously circulating CD34+ cells have been exploited for autologous backup, to be reinfused in case of graft failure. Although HSPC mobilization represent an important issue for the feasibility of gene therapy, mobilization of osteopetrotic patients remains a poorly investigated approach. Few anecdotal reports of drug-induced mobilization in osteopetrosis are available, suggesting its feasibility at least in patients with low levels of circulating CD34+ cells (17, 18).

In this work, we showed the proof-of-efficacy of lentiviral vector GT for osteopetrosis in restoring osteoclast resorption activity in the *oc/oc* murine model, using a clinically-optimized lentiviral vector. Moreover, we modeled the tools to support a feasible GT and to reduce the burden of transplant-related procedures in ARO patients. First, we provided evidence of the effect of HSPC mobilization in neonate *oc/oc* mice, which represents an instrumental model to study transplantation issues in the pathological *TCIRG1*-defective BM niche. Second, we demonstrated that antibody-drug conjugates may represent a valid option for non-genotoxic pre-transplant conditioning for osteopetrosis.

## Materials and methods

### Mice

Animal experimental procedures were approved by the Institutional Animal Care and Use Committee of San Raffaele Hospital and Italian Ministry of Health. C57BL/6 wild-type (WT) CD45.2 and CD45.1 mice (strain codes 027 and 494, respectively) were obtained from Charles River Laboratories (Calco, Italy). The *oc/oc* mouse strain (7) (The Jackson Laboratories strain no. 000230) was fully backcrossed to C57BL/6 background before the start of experiments and maintained on site in heterozygosis.

### Mouse genotyping

Mice were genotyped by PCR using DNA extracted from a tail biopsy, using Extract-N-Amp™ Tissue PCR Kit (Sigma Aldrich, St. Louis, MO, USA). The PCR conditions to detect *Tcirg1* mutation were: 95°C for 5 min, then 40 cycles, 95°C for 30 s, 60°C for 30 s and 72°C for 30 sec, then 72°C for 5 min, with *oc/oc* forward primer 5′-GGCCTGGCTCTTCTGAAGCC-3′, *oc/oc* reverse primer 5′-CCGCTGCACTTCTTCCCGCA-3′, WT forward primer 5’-TCATGGGCTCTATGTTCCGG-3’ and WT reverse primer 5’-GAAGGCGCTCACGGATTCGT-3’. WT mice present a PCR product of 431 bp, while *oc/oc* mice display a 563 bp PCR product. Heterozygous mice have both products.

### Lentiviral vector production

PGK.TCIRG1 plasmid and lentiviral vector were produced as previously described (13). Briefly, VSV-pseudotyped third-generation LVs were produced by cotransfection of the transfer, packaging (pMD2.Lg/p.RRE and pRSV.Rev) and envelope constructs (pMD2.G) into 293T cells by a Ca_3_PO_4_ transfection. Supernatants were collected, passed through a 0.22 μm filter, and purified by ultracentrifugation. Vector titer (transducing units/ml) was estimated by digital droplet PCR (ddPCR) (Bio-Rad, Hercules, CA, USA) on 293T cells, previously infected with different dilutions of concentrated vector supernatant.

### Lin- isolation and transduction

Donor WT and *oc/oc* mice were euthanized at post-natal day (PND) 12 by decapitation and spleen was collected. Single cell suspension was obtained by smashing on 40 μm mesh to collect splenocytes for Lineage-negative (Lin–) separation. Alternatively, pregnant mice were euthanized on post-coitum day 14.5. Fetal livers (FLs) were dissected out of embryos and smashed to obtain a single-cell suspension. Lin- cells were enriched from total splenocytes or FL cells with the Lineage Cell Depletion Kit (Miltenyi Biotec, Bergisch Gladbach, Germany), according to the manufacturer’s instructions. *oc/oc* Lin- cells were transduced overnight with LV_PGK.TCIRG1 at a multiplicity of infection (MOI) of 10 in StemSpan SFEM medium (STEMCELL Technologies, Vancouver, Canada), 1% penicillin/streptomycin, 1% glutamine (Gibco, Waltham, MA, USA), and the following cytokines (PeproTech, Rocky Hills, NJ, USA): recombinant murine thrombopoietin (rmTPO), 20 ng/mL; recombinant murine stem cell factor (rmSCF), 50 ng/mL; recombinant human Fms-related tyrosine kinase 3 ligand (rhFLT3L), 10 ng/mL; recombinant human interleukin 3 (rmIL-3), 10 ng/mL; and recombinant human interleukin 6 (rhIL-6), 20 ng/ml. *oc/oc* transduced and WT Lin- were frozen alive in fetal bovine serum (FBS) (Euroclone, Milan, Italy) supplemented with 10% dimethyl sulfoxide (DMSO) and stored at -80°C until use.

### *oc/oc* mice transplantation

In GT experiments, recipient *oc/oc* mice were conditioned on post-natal day 1 or 2 by sublethal total-body irradiation (300 rad), at least 2 hours before transplantation. Then, they were injected intra-hepatically with 0.66-1.4*10^6^ Lin- cells (*oc/oc* Lin- transduced with LV_PGK.TCIRG1 or WT Lin-). In CD45-saporin (SAP) experiments, recipient *oc/oc* mice were conditioned by CD45-SAP injection or total body irradiation (IRR), as indicated. They were then injected intra-hepatically with 7*10^6^ total BM cells, collected from adult WT CD45.1 mice.

Gentamicin sulphate (Italfarmaco, Milan, Italy) was administered in drinking water (8 mg/mL) for the first 2 weeks after transplantation to prevent infections. 18-20 days after transplant mice were weaned and fed with DietGel Boost (ClearH_2_O, Westbrook, ME, USA) due to the absence of teeth. Mice were checked for health status and weight weekly until the end of the experiment, set at 4 months after GT or earlier in case of profound weight loss. Splenocytes, BM cells, bones, blood and thymus were collected for analysis.

### Osteoclasts differentiation

OCs were generated from splenic or FL Lin- cells *in vitro*, differentiating them toward myeloid lineage in StemSpan SFEM medium (STEMCELL Technologies, Vancouver, Canada), 2% FBS (Euroclone, Milan, Italy), 1% penicillin/streptomycin, 1% glutamine (Gibco, Waltham, MA, USA), and the following cytokines (PeproTech, Rocky Hills, NJ, USA): rmTPO, 20 ng/mL; rmSCF, 50 ng/mL; rhFLT3L, 10 ng/mL; rmIL-3, 10 ng/mL; and rhIL-6, 20 ng/mL for at least 10 days, splitting cells every 3 days. As previously described (19, 20), OC precursors from differentiated Lin-, BM and splenic cells were seeded on tissue-culture treated 96-well plates or dentine slices (Immunodiagnostic Systems, East Boldon, United Kingdom) at a density of 5*10^5^ in 200 μl of alpha-Minimum Essential Medium (Sigma-Aldrich, St. Louis, MO, USA) containing 10% FBS, 1% penicillin, 1% streptomycin and the following cytokines: recombinant murine macrophage colony-stimulating factor (rmM-CSF), 25 ng/ml; recombinant human transforming growth factor-beta (rhTGFβ), 5 ng/ml; dexamethasone, 1 μM (Sigma-Aldrich, St. Louis, MO, USA); recombinant murine receptor activator of nuclear factor kappa-B ligand (rmsRankL), 100 ng/ml. Half of the medium was chanced twice a week for at least 7 days for OCs seeded on plastic 96-well plates and for 21 days for OCs seeded on dentine slices. Cells were incubated at 37°C and 5% CO_2_.

C-telopeptide fragments from type I collagen (CTX-I), released during resorption assay, were quantified using CrossLaps for Culture (CTX-I) ELISA (Immunodiagnostic Systems, East Boldon, United Kingdom).

### Osteoclast activity

OCs cultured on plastic were stained using the Tartrate Resistant Acid Phosphatase (TRAP) Kit (Sigma-Aldrich, St. Louis, MO, USA), following the manufacturer’s instruction. To evaluate OC resorptive function, OCs were differentiated on dentin discs (Immunodiagnostic Systems, East Boldon, United Kingdom). After 3 weeks, dentine discs were rinsed with water, scraped to remove attached cells, stained with 1% toluidine blue solution for 3 minutes, and then washed with water to visualize resorption pits. TRAP and toluidine blue images were acquired on a Zeiss AxioImager microscope.

### Colony forming unit assay

Hematopoietic progenitor cultures were performed plating Lin- cells or total cells from blood, spleen, BM and liver in MethoCult GF M3434 methylcellulose-based medium (STEMCELL Technologies, Vancouver, Canada) and cultured for 12 days.

### Parathormone ELISA assay

Serum samples were collected from mice and stored at -20°C until use. Mouse PTH 1-84 ELISA Kit (Quidel Corporation, San Diego, CA, USA) was used to evaluate PTH concentration in the serum samples, following the manufacturer’s instruction.

### Flow cytometry

Single-cell suspensions from tissues were obtained smashing tissues through a 40 µm cell strainer. Red blood cells of peripheral blood were lysed with ACK lysing buffer (Gibco, Waltham, MA, USA) to obtain white blood cells (WBCs). WBC count was determined with Procyte DX Analyser (IDEXX laboratories, Westbrook, ME, USA). Single-cell suspensions and WBCs were stained for 15 min at room temperature with the following antibodies from BD Pharmingen (Franklin Lakes, NJ, USA), Miltenyi Biotec (Bergisch Gladbach, Germany), BioLegend (San Diego, CA, USA) or eBioscience (San Diego, CA, USA): CD3, CD4, CD8, CD11b, CD19, CD21, CD23, CD24, CD25, CD44, CD45.1, CD45.2, CD48, CD117, CD150, B220, Lin+ cocktail, and Sca1. Viability was determined by using the Live/Dead Fixable Dead Cell Stain Kit (Thermo Fisher Scientific, Waltham, MA, USA). Samples were acquired on a FACSCanto II and FACSymphony A5 (BD Biosciences, Franklin Lakes, NJ, USA) and analyzed with FlowJo software.

### Histopathology

Spleen, brain and kidney were fixed in 10% neutral buffered formalin for 24 hours, washed and maintained in ethanol 70% at 4°C. Bone samples were fixed in 4% paraformaldehyde (PFA) and decalcified in 14% ethylenediaminetetraacetic acid (EDTA), pH 7.4 with Acetic Acid. Then samples were routinely processed for histology and embedded in paraffin. Four micrometers thick paraffin sections were stained with hematoxylin and eosin (H&E), TRAP, and avidin-biotin complex immunohistochemistry with a rabbit polyclonal anti-cleaved caspase-3 primary antibody (Cell Signaling Technology, Danvers, MA, USA). Slides were then evaluated at the light microscope. Images of H&E and cleaved caspase-3 immunolabeled sections were acquired on a Leica DM2500 microscope equipped with a Leica DFC310 FX camera. Morphometric analyses of spleen were performed using Olympus Slide Scanner VS120-L100 to acquire digital images and Image-pro software to evaluate the white to red pulp ratio.

### Micro-computed tomography

Femurs and skull were fixed in 10% neutral buffered formalin for 24 hours at 4°C. Then formalin was replaced with 70% ethanol for 24 hours and finally with 80% ethanol. The spine was fixed in 4% paraformaldehyde solution for 24 hours at 4°C and then stored in PBS. For micro-CT analysis, the femur and skull of each mouse were placed into a radiotranslucent sample holder. To prevent desiccation, the holder was filled with PBS (Life Technologies, Carlsbad, CA, USA). Micro-CT scanning and analysis of femurs and skull was performed as previously described (21) using a micro-CT 40 desktop cone-beam micro-CT (Scanco Medical AG, Bruüttisellen, Switzerland). Micro-CT scanning was performed with a voxel size of 10 µm or 14 µm for femora and skull, respectively (1000 projections per slice with 2048 samples and 200-s sample time at a tube energy of 55 kVp with an intensity of 145 µA). Reconstructed slices were inspected using the Scanco MicroCT software suite.

For the analysis of vertebrae, the lumbar area of the vertebral column was wrapped in gauze moistened with PBS and enclosed in parafilm. The micro-CT acquisition was performed at 7 µm with a SkyScan 1276 (Bruker, Kontich, Belgium) at 55 kV and 72 µA, using a 0.25 mm Aluminium filter. The reconstruction was carried out with the SkyScan NRecon software (InstaRecon reconstruction engine), applying ring artifact reduction and beam hardening correction. Image analysis of the L5 vertebra was performed with a semiautomatic approach, developed in house with the Skyscan CT Analyser software (CTAn), to segment the trabecular region of the vertebral body in the coronal plane. The bone mineral density (BMD) of the trabecular region was calculated by resorting to a calibration based on a linear extrapolation against mean attenuation coefficients from selected regions of a 2 mm phantom rod pair containing 0.25 and 0.75 g/cm^3^ CaHA (Skyscan). A 3D morphometric analysis of the trabecular region was finally performed after binarization of the trabeculae by an adaptive thresholding algorithm.

### Vector copy number

Genomic DNA was extracted with QIAamp DNA Blood mini kit (QIAGEN, Hilden, Germany), according to manufacturer’s instructions. Vector copy number/genome (VCN) was quantified by digital droplet PCR using murine RPP30 housekeeping gene, as previously described (22).

### Plerixafor-based HSPC mobilization

*oc/oc* and WT mice at PND 3 or 7 were injected subcutaneously with 5 mg/kg Plerixafor (Mozobil from Genzyme, Cambridge, MA, USA). A cohort of *oc/oc* and WT mice remained untreated as control group. One hour after the injection, mice were euthanized, and the blood was collected in vials containing 50 µl citrate phosphate dextrose solution as anticoagulant. WBC count was determined with Procyte DX Analyser (I IDEXX laboratories, Westbrook, ME, USA).

### Non genotoxic antibody-drug conjugate conditioning

The CD45-SAP immunotoxin was prepared as described (23) by combining a biotinylated anti-CD45.2 antibody (clone 104, Biolegend, San Diego, CA, USA) with streptavidin-SAP conjugate (Advanced Targeting Systems, San Diego, CA, USA) in a 1:1 molar ratio. The CD45-SAP immunotoxin was injected in the temporal vein at birth, while control group was injected con PBS. Irradiated group was not injected and received total body irradiation (300 RAD) on PND 2. In transplantation experiments, animals were transplanted 48 hours after the injection or 2 hours after IRR, receiving 7*10^6^ CD45.1 total BM cells by intra-hepatic injection. For HSPC depletion experiment, mice were euthanized 48 hours after the CD45-SAP/PBS injection or 2 hours after IRR.

### Statistical analysis

Values are expressed as means ± standard deviation (SD) or standard error of the mean (SEM), as indicated in figure legends. Non-parametric one-way ANOVA (Kruskall-Wallis with Dunn’s multiple comparison test) and Log-rank test were performed as indicated in figure legends, using GraphPad Prism v.10.2.2 software. p values are shown as follows: *p < 0.05; **p < 0.01; ***p < 0.001; ****p < 0.0001.

### Data availability

All data needed to evaluate the conclusions in the paper are present in the paper and/or the **Supplementary Materials**. Raw data will be made available upon reasonable request.

## Results

### *In vitro* correction of *oc/oc* osteoclasts

We generated a clinically-optimized LV carrying *TCIRG1* cDNA under the control of phosphoglycerate kinase (PGK) promoter (LV_PGK.TCIRG1) (**Figure 1A**), that efficiently corrects the bone resorptive function of ARO patient osteoclasts (OCs) *in vitro* (13). We set up a protocol for the *in vitro* differentiation and correction of murine *oc/oc* OCs, starting from splenic lineage-negative (Lin-) cells (**Figure 1B**). Since *oc/oc* mice show a severe osteopetrotic phenotype and a life expectancy of less than 3 weeks, Lin- cells were isolated from splenocytes of *oc/oc* mice on post-natal day (PND) 12. Lin- cells were transduced overnight with LV_PGK.TCIRG1 and cultured in the presence of a cytokine cocktail to drive myeloid commitment and then OC differentiation. We observed a mean VCN of 18.1 ± 4.4. As expected, *oc/oc* cells differentiated into multinucleated tartrate-resistant acid phosphatase (TRAP)-positive OCs independently of the transduction and comparably to WT control (**Figure 1C**, upper panels). However, unlike untransduced (UT) *oc/oc* OCs, LV_PGK.TCIRG1 transduced *oc/oc* cells generated functional OCs, able to resorb dentin slices (**Figure 1C**, lower panel). At the end of the culture, we quantified the C-telopeptide degradation products from type I collagen (CTX-I) released in the supernatant during resorption of dentine discs by osteoclasts. We observed comparable CTX-I levels released by WT and *oc/oc* GT osteoclasts, while *oc/oc* untreated cells are completely unable to resorb dentine discs (**Figure 1D**).

**Figure 1:**
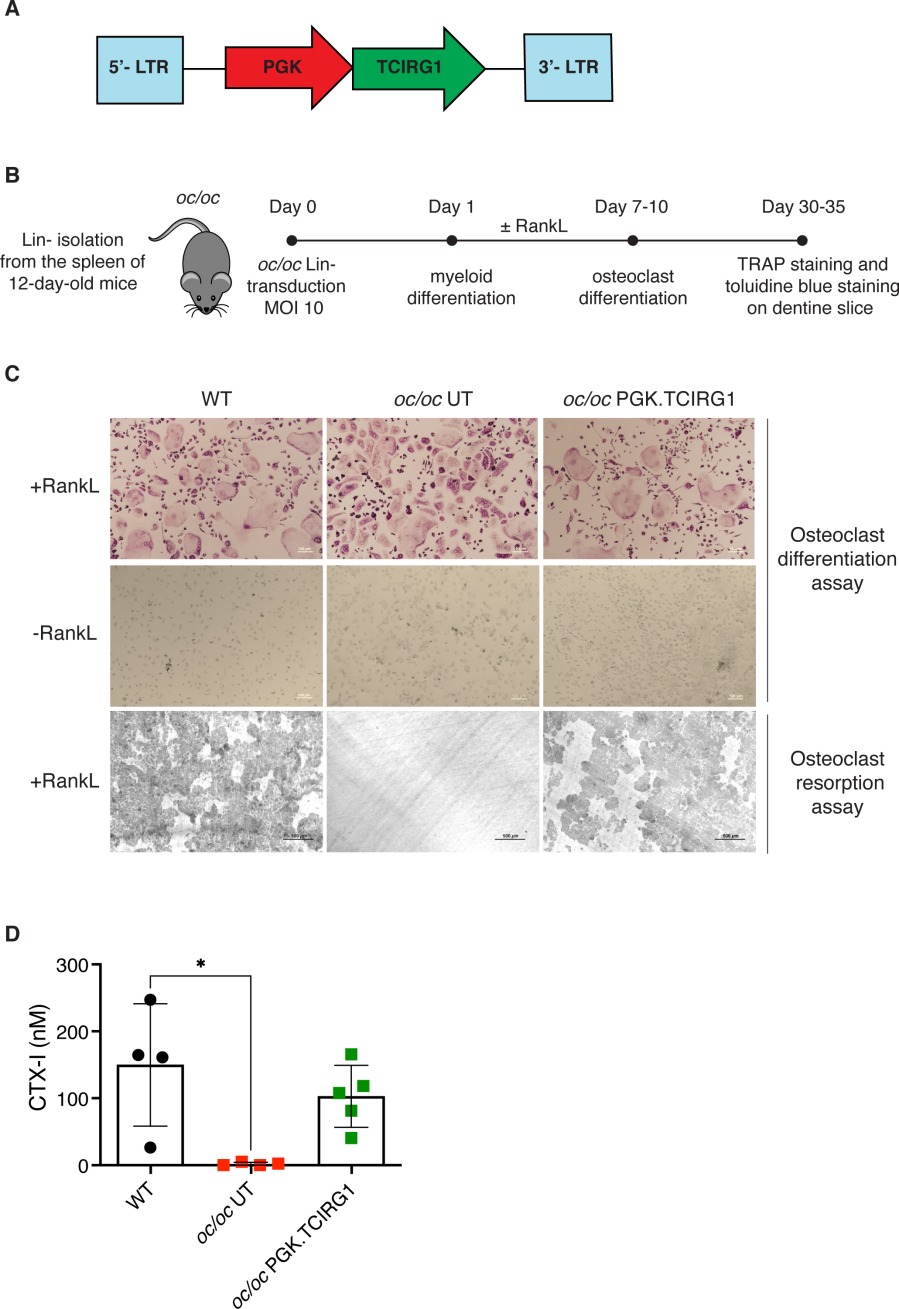
*In vitro* gene therapy (GT) of *oc/oc* derived osteoclasts (OCs). **(A)** Scheme of the lentiviral vector (LV) LV_PGK.TCIRG1, containing the human *TCIRG1* cDNA under the control of the phosphoglycerate kinase (PGK) promoter, flanked by defective long terminal repeat elements (LTR). **(B)** Experimental scheme: splenic Lin- cells are isolated from 12-day-old pups and transduced with LV_PGK.TCIRG1 at a multiplicity of infection (MOI) 10. Then cells are differentiated towards the myeloid lineage for 7-10 days. Once myeloid committed precursors are obtained, OC differentiation is induced to evaluate differentiation and bone resorption capacity. **(C)** Representative picture of OCs differentiated from WT, untransduced (UT) *oc/oc* and LV_PGK.TCIRG1-transduced *oc/oc* Lin- cells, cultured in presence (top) or absence (middle) of RankL cytokine. OCs were stained for tartrate-resistant acid phosphatase (TRAP) activity. The bottom panel shows representative picture of bone resorption assay performed on OCs cultured on dentine slices and stained with toluidine blue to visualize bone resorption pits (darker areas). All images were acquired with Nikon ECLIPSE E600 microscope equipped with Nikon DS-Ri2 camera, using Plan Fluor 10x/0.13 objective and NIS-Elements F 4.30.01 software. **(D)** Quantification of C-telopeptide fragments from type I collagen (CTX-I) in the culture supernatant, released during resorption assay, by functional osteoclasts derived from WT, *oc/oc* untransduced (UT) and *oc/oc* PGK.TCIRG1-transduced Lin- cells.

### Gene therapy improves survival and phenotype of *oc/oc* mice

To test the efficacy of our *ex vivo* GT protocol, we also exploited fetal liver as a source of Lin- cells, to avoid the frequent perinatal loss and increase the number of available donor *oc/oc* mice. After isolation, Lin- cells were transduced with LV_PGK.TCIRG1 and transplanted by intra-liver injection into irradiated *oc/oc* neonates at PND1 or 2 (**Figure 2A**). We followed GT mice overtime up to 16-20 weeks after treatment. Ten out of 14 GT mice overcame the expected *oc/oc* life span (less than 3 weeks), and 6 of them reached the age of 4-5 months (**Figure 2B**). In parallel, we transplanted UT WT Lin- cells in 15 *oc/oc* recipients (*oc/oc* Tx Lin- WT), as positive control. Half of *oc/oc* Tx Lin- WT survived longer than 3 weeks and 4 of them were still alive at the end of the experiment (**Figure 2B**). Survival rate was comparable in the 2 treatment groups and demonstrated the non-inferiority of GT protocol versus conventional HSCT from WT donors. Similarly to *oc/oc* Tx Lin- WT mice, GT mice showed absence of circling behavior and restored, though abnormal, eruption of the incisors (**Figure 2C**). However, they remained smaller than WT littermates and WT-transplanted controls in body weight and size (**Figures 2C, D**).

**Figure 2:**
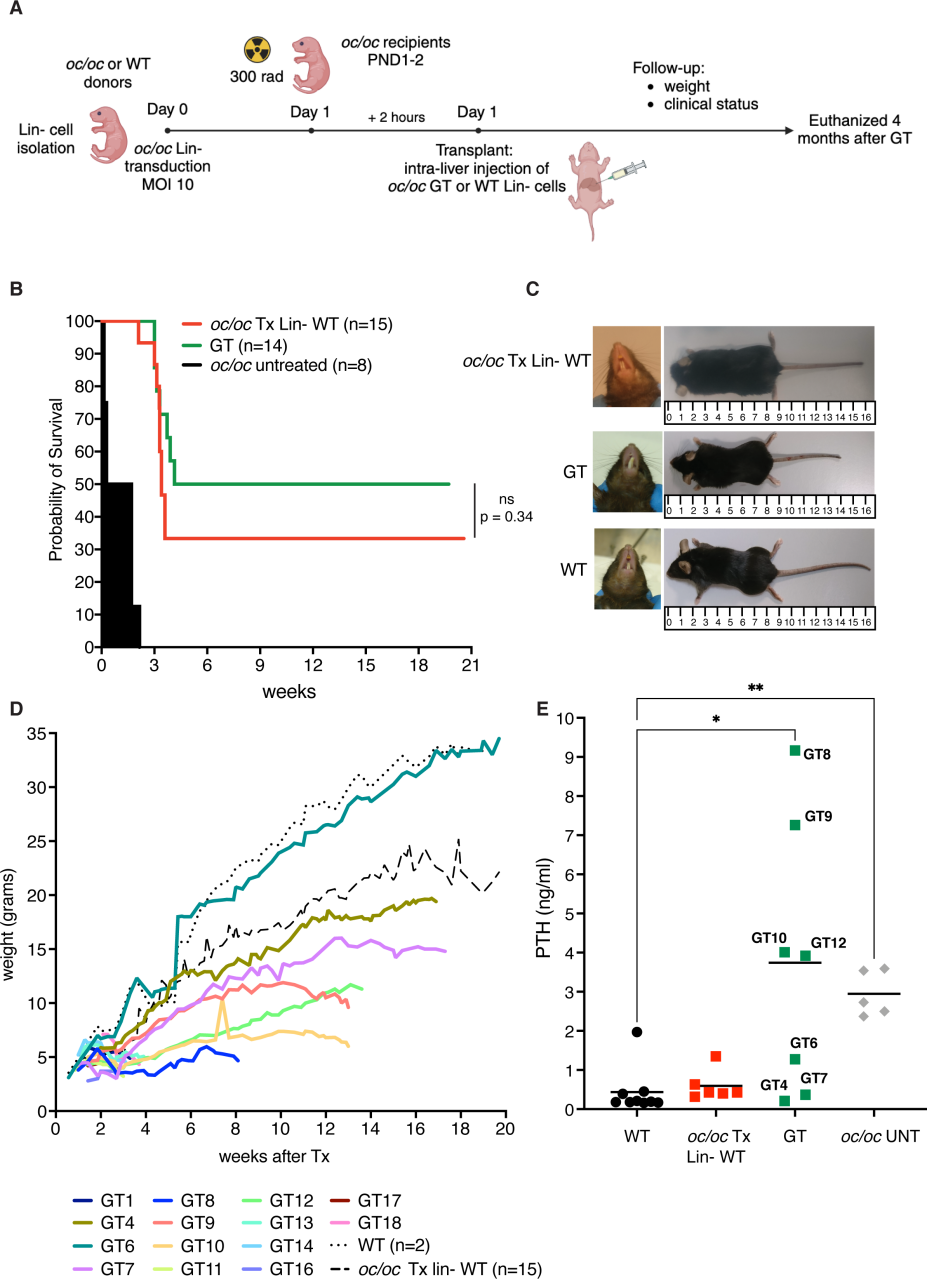
*Ex vivo* gene therapy (GT) of *oc/oc* mice. **(A)** Experimental scheme: Lin- cells are isolated from 14.5 days-post-coitum fetal livers of *oc/oc* embryos and transduced with LV_PGK.TCIRG1 at a multiplicity of infection (MOI) of 10. Cells are then transplanted by intra-liver injection into 300 rad conditioned *oc/oc* recipients at post-natal day (PND) 1 or 2. Transplanted mice were followed overtime for their clinical status and weight. The end of the experiment was set at 4 months after the transplant. Created with BioRender.com. **(B)** The Kaplan-Meier curve shows the survival of GT mice (GT, n=14) compared to *oc/oc* mice transplanted with WT Lin- cells (*oc/oc* Tx Lin- WT, n=15) and *oc/oc* untreated mice (n=8). Statistical analysis: Log-rank test, ns = not significant, p value is indicated. **(C)** Representative pictures show incisor teeth and body size of GT mice compared to *oc/oc* transplanted with WT Lin- cells and WT littermates. **(D)** The graph shows in color the body weights of all GT mice in comparison to the mean of *oc/oc* Tx Lin- WT and WT controls overtime (in black dashed and dotted lines, respectively). **(E)** Parathormone (PTH) concentration (ng/ml) in the serum of indicated experimental groups. Each dot represents the mean of two technical replicates. Labels indicate the identification numbers of GT mice. Serum of *oc/oc* transplanted with WT Lin- (n=6), GT mice (n=7) and WT controls (n=9) was collected at termination (8-20 weeks of age), while serum from *oc/oc* untreated (UNT) controls (n=5) was collected at 2-3 weeks of age. Statistical analysis: non-parametric one-way ANOVA with Dunn’s multiple comparison post-test. *p<0.05, **p<0.01.

At termination, we collected peripheral blood, bones and hematopoietic organs (bone marrow, spleen and thymus). To have an indirect measurement of bone resorption *in vivo*, we measured the level of parathormone (PTH) in the serum of GT mice. The osteopetrotic phenotype includes metabolic defects related to calcium homeostasis, due to the absent bone resorptive activity of OCs. In particular, hypocalcemia is coupled with hyperparathyroidism in *oc/oc* mice, due to a compensatory mechanism (8). In some GT mice (namely GT4, GT6, GT7), we observed PTH concentrations comparable to WT and *oc/oc* Tx Lin- WT controls. However, other mice such as GT8, GT9, GT10 and GT12 showed a high PTH concentration in the serum, more similar to *oc/oc* untreated mice, despite their long survival and their good clinical status (**Figure 2E**).

### GT ameliorates bone architecture

Bone geometry was evaluated by microcomputed tomography (micro-CT). Femora of GT4 and GT6 mice showed bone architecture and cortical thickness comparable to both the WT untreated control and to the *oc/oc* mice transplanted with WT Lin- cells, suggesting a full reconstitution of the bone phenotype, in line with PTH levels. Conversely, GT7, GT8, GT9 and GT10, despite the significant improvement of the lifespan, displayed an impaired bone structure, typical sign of osteopetrosis (**Figure 3A**). This was confirmed by micro-CT quantification of bone mineral density (BMD), bone volume fraction (% BV/TV) and bone length (**Figures 3B–D** and **Supplementary Figure 1**). Analysis of the skull displayed the restored eruption of the incisors in all GT mice but GT7. In line with the results obtained in the femora, GT6 skull appeared comparable to WT and *oc/oc* Tx Lin- WT mice. On the other hand, skulls of GT8, GT9 and GT10 showed the typical “moth-eaten” appearance due to thinned cortical bone and increased cortical porosity (**Figure 3E**), suggesting the persistence of osteopetrorickets (8, 24, 25) caused by partial correction of the bone phenotype after GT and by the altered calcium uptake in the stomach (8).

**Figure 3:**
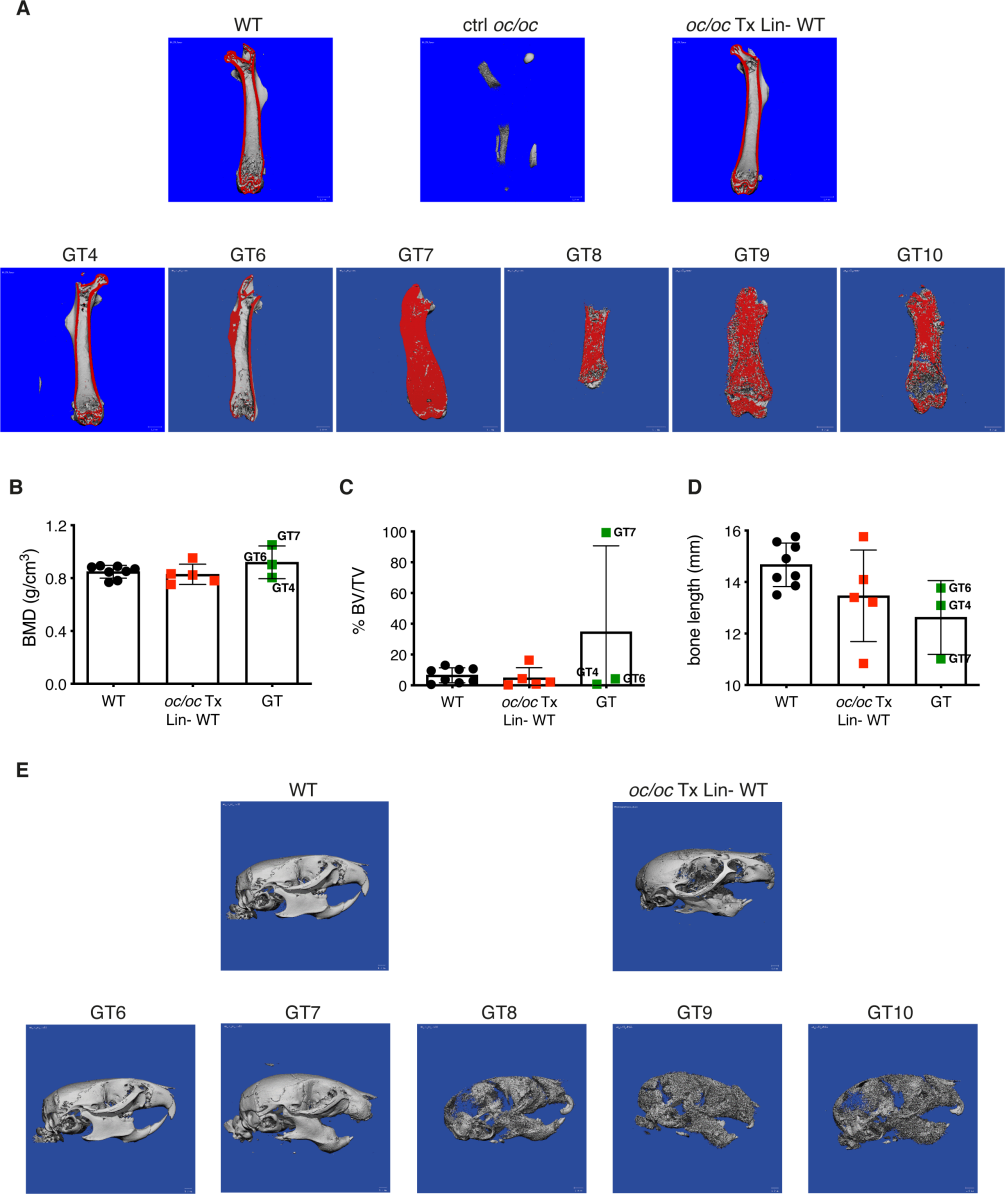
Bone structure analysis by micro-computed tomography (micro-CT). **(A)** Representative images showing micro-CT scanning of the femur of adult GT mice compared to *oc/oc* mice transplanted with WT Lin- cells and WT littermates. The cortical area of the bone is highlighted in red. Note that the cortical bone is clearly distinguished in GT4 and GT6, as well as in WT and *oc/oc* Tx Lin- WT mice. On the contrary, the bone was still extremely dense in GT7-GT10 and the cortical and trabecular components could not be distinguished. Image of *oc/oc* have been obtained from a 2-week-old mouse, but the dimension is inadequate for micro-CT. GT12 mouse: not done. **(B)** Bone mineral density (BMD) quantification in the trabecular region of the femur, performed on the images acquired in panel **(A)** GT8: scanned, not possible to quantify. GT9 and GT10: scanned but not quantified. GT12: not done. **(C)** Bone volume fraction (% BV/TV): ratio of the segmented bone volume to the total volume of the region of interest **(D)** Bone length (mm) of the scanned femurs. **(E)** Representative images of the skull of adult GT mice compared to *oc/oc* mice transplanted with WT Lin- cells and WT littermates. Labels indicate the identification numbers of GT mice. GT4 and GT12: not done.

For in depth evaluation of the bone architecture, the femur and the vertebral column of GT mice were analyzed by histology. As expected, untreated *oc/oc* vertebrae, collected from 2- or 3-week-old mice, exhibited impaired trabecular bone organization and very limited BM space (**Figure 4A**). GT mice showed a variable reconstitution of physiological bone architecture, spanning from full rescue in GT4 and GT6 to dense bones with small clusters of hematopoietic cells in GT1, GT7, GT9, GT10 and GT12 (**Figure 4A**). Femora sections were stained for TRAP, an osteoclast marker. Coherently, GT4 and GT6 mice showed TRAP-positive bone-resorbing OCs in the growth plate, as well as in cortical and trabecular bones, with absence of BM fibrosis. Conversely, GT1, GT7, GT9 and GT10 showed diffuse fibrosis of bones, which caused nonspecific TRAP signal (**Figure 4B**).

**Figure 4:**
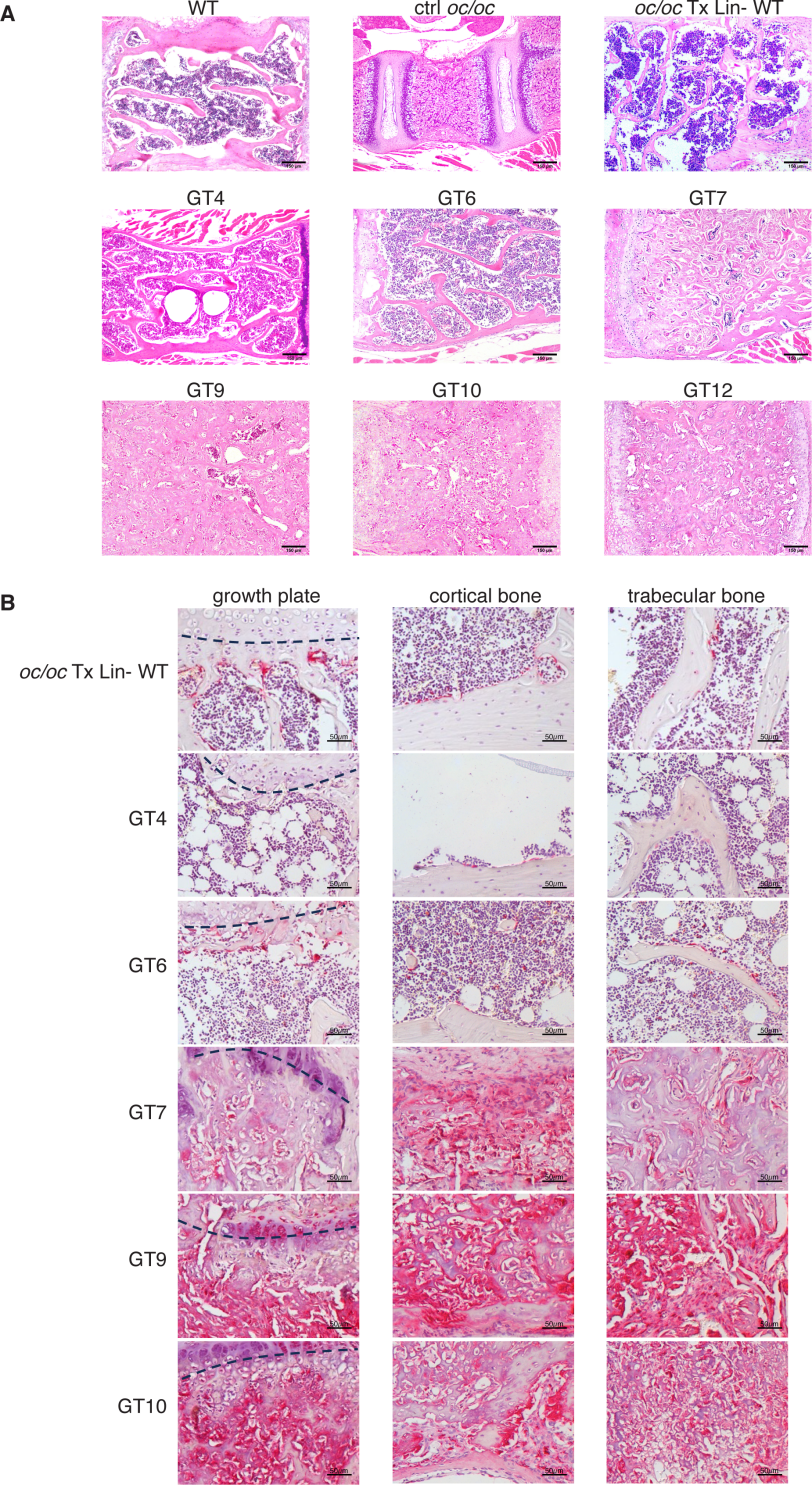
Histological analysis of vertebral column and femur. **(A)** Representative images of hematoxylin and eosin (H&E) staining of vertebral column section of GT compared to WT and *oc/oc* untreated controls and *oc/oc* mice transplanted with WT Lin- cells. Note that WT, *oc/oc* Tx Lin- WT and GT were obtained from adult mice (8-20 weeks of age), while *oc/oc* untreated controls were 2-week-old. Images were acquired with a 100x magnification (Leica DM2500 microscope equipped with Leica DFC310 FX camera). GT8: not done. **(B)** Images show tartrate-resistant acid phosphatase (TRAP) staining of femora sections of *oc/oc* mice transplanted with WT Lin- or GT cells. Images were acquired with a 200X magnification of the growth plate (left, the growth plate is outlined with a dashed line), in the cortical (middle) and in the trabecular (right) areas of the bone. GT8 and GT12: not done.

### Bone reconstitution positively impacts on hematopoiesis efficiency

Next we assessed the cellularity and immune composition of the hematopoietic organs, since *oc/oc* mice exhibit very low BM cellular content and a B cell defect secondary to the osteoclast defects (26, 27).

Total BM cellularity in GT mice was similar to WT control and *oc/oc* Tx Lin- WT mice (**Figure 5A**), and higher than 3-week-old *oc/oc* untreated mice, in which BM cells are nearly absent (26). The BM counts of GT mice reflected the bone architecture, with normal counts in GT4, GT6 and GT12 animals and lower in the others (**Figure 5A**). Concordantly, frequency and counts of B cells in GT4 and GT6 were comparable to WT and *oc/oc* Tx Lin- WT mice, whereas the remaining GT mice showed low B cell frequency (**Figures 5B, C**). Similar results were obtained for myeloid and hematopoietic stem cells (HSCs) in the BM (**Supplementary Figures 2A–D**). No major defects were observed in the colony forming unit (CFU) assay from the BM, suggesting a normalization in the number and output of hematopoietic stem and progenitor cells (HSPCs) (**Figure 5D**).

**Figure 5:**
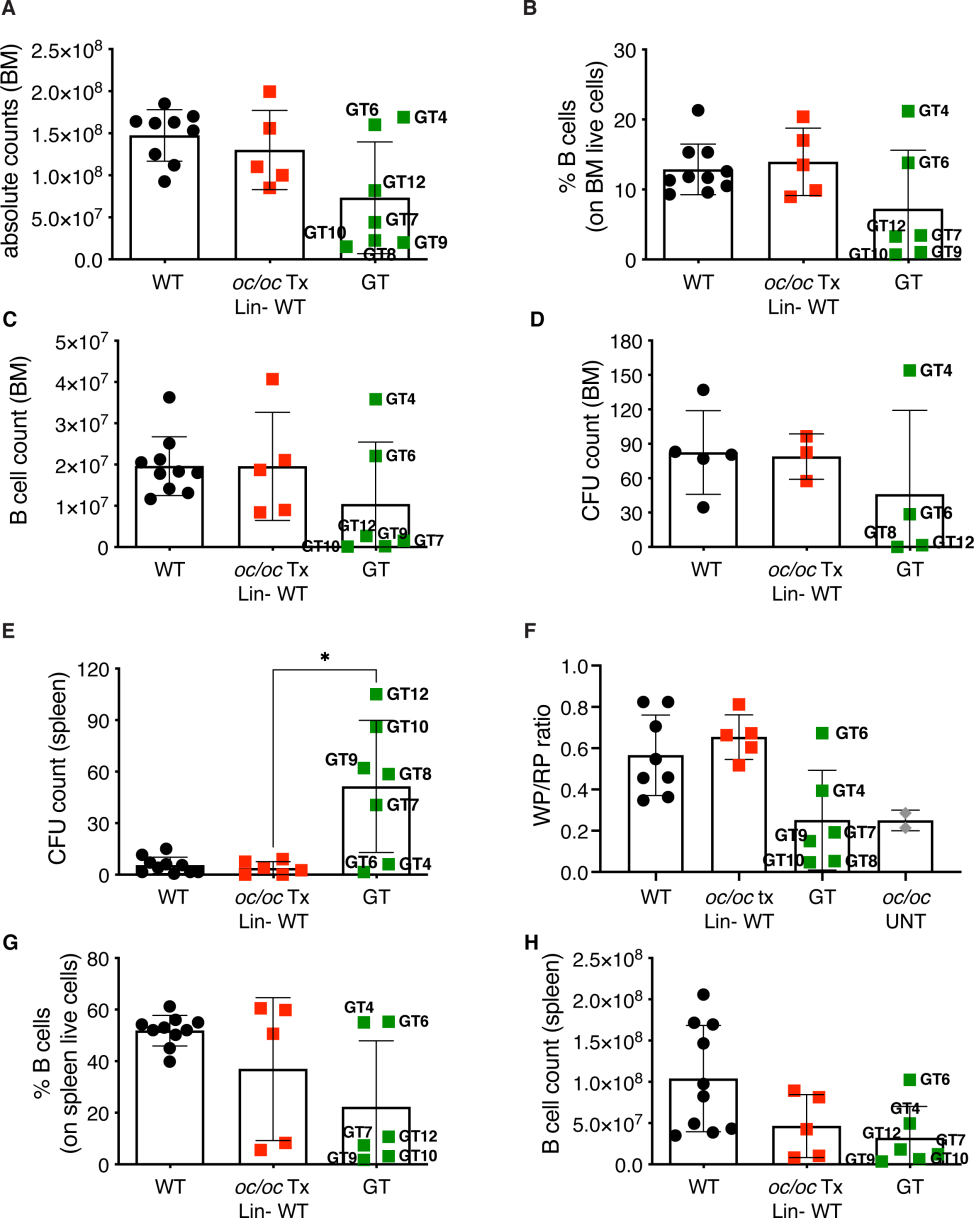
Hematopoietic organ readouts. **(A)** Total bone marrow (BM) cellularity in WT, *oc/oc* Tx Lin- WT and GT mice. Labels indicate the identification numbers of GT mice. **(B)** Frequency of B cells in the BM. GT8: not done. **(C)** Absolute counts of B cells in BM. **(D)** Counts of colony forming units (CFUs) from total BM. GT7, GT9 and GT10: not available due to culture contamination. **(E)** Counts of colony forming units (CFUs) from splenocytes. **(F)** Ratio of the white and red pulp areas (WP/RP) of the spleen. WP/RP of oc/oc untreated controls (UNT) is also reported. GT12: not done. **(G)** Frequency of B cells in the spleen. GT8: not done. **(H)** Absolute counts of B cells in spleen. Bars indicate mean ± SD. Statistical analysis: non-parametric one-way ANOVA with Dunn’s multiple comparison post-test. *p<0.05.

Conversely, we observed increased CFU counts from splenocytes of most GT animals (**Figure 5E**), with the exception of GT4 and GT6, in spite of normal total cell counts of the spleen (**Supplementary Figure 3A**), suggesting the persistence of compensatory extramedullary hematopoiesis after GT. Histological analysis of the spleen showed abnormal distribution of white and red pulp (**Supplementary Figure 3B**) and a decreased white/red pulp ratio in GT mice except for GT4 and GT6 (**Figure 5F**). Also in this organ, B cell frequency and counts after GT were generally low, apart from GT4 and GT6 (**Figures 5G, H**). Low absolute counts were observed in all B cell subsets of the spleen, namely follicular, marginal zone and transitional cells, while the proportion of the different subsets appeared comparable to controls (**Supplementary Figures 4A–F**). On the other hand, no changes were detected in T cells (**Supplementary Figures 4G, H**). Similar results were observed in peripheral blood with decreased B cell frequency and counts in most GT animals, but GT4 and GT6, and no changes in the T or myeloid compartments (**Supplementary Figures 5A–F**). Notably, total white blood cell count was comparable to controls (**Supplementary Figure 5G**), indicating the resolution of pancytopenia, common in ARO patients. In line with PB and spleen, absolute counts and T cell subset proportions in the thymus were normal after GT (**Supplementary Figure 6**).

Despite the long-term survival, in most GT mice we found low vector copy number/genome (VCN) in the hematopoietic organs (BM, spleen and thymus) and in the bulk CFU population from BM and spleen (**Table 1**). We hypothesized that loss of the engraftment overtime may have occurred. However, few corrected HSPCs and long-lived osteoclast syncytia could possibly sustain bone remodeling and long-term survival of GT mice. We performed RNAscope, an *in situ* hybridization assay, to detect transgene expression from the LV in the splenic tissue of GT mice (**Supplementary Figure 7**). We detected lentiviral expression in all GT mice at variable levels. Higher expression was observed in mice that survived longer than 16 weeks (GT4, GT6, GT7, GT9), while few positive cells were visible in mice that died between 8- and 14-weeks post GT (GT8, GT10, GT11).

**Table 1:** Data from individual GT mice transplanted with LV_PGK.TCIRG1 transduced *oc/oc* Lin-.

mouse ID	Lin- source	transplanted cells (no.)	*In vitro* VCN of transplanted Lin- cells	survival (weeks)	*In vivo* VCN			VCN of MethoCult pool	
					SP	BM	THY	SP	BM
GT1	spleen	0.7*10^6^	31.4	3	n.d.	n.d.	n.d.	n.d.	n.d.
GT4	spleen	1.0*10^6^	31.4	17	4.11	4.69	5.00	12.00	6.21
GT6	fetal liver	2.1*10^6^	15.6	20	0.000	0.013	0.000	0.500	0.008
GT7	fetal liver	0.7*10^6^	n.d.	17	0.008	0.009	0.000	0.000	n.d.
GT8	fetal liver	0.7*10^6^	n.d.	8	0.003	0.004	0.006	0.002	n.d.
GT9	fetal liver	1.2*10^6^	n.d.	14	0.004	0.006	0.039	0.002	n.d.
GT10	fetal liver	1.2*10^6^	n.d.	14	0.005	0.001	0.022	0.028	n.d.
GT11	fetal liver	1.4*10^6^	n.d.	3	n.d.	n.d.	n.d.	n.d.	n.d.
GT12	fetal liver	1.3*10^6^	1.71	16	0.006	0.069	0.008	0.000	0.004
GT13	fetal liver	0.9*10^6^	n.d.	4	n.d.	n.d.	n.d.	n.d.	n.d.
GT14	fetal liver	0.9*10^6^	n.d.	4	n.d.	n.d.	n.d.	n.d.	n.d.
GT16	fetal liver	1.2*10^6^	n.d.	3	n.d.	n.d.	n.d.	n.d.	n.d.
GT17	fetal liver	1.2*10^6^	n.d.	3	n.d.	n.d.	n.d.	n.d.	n.d.
GT18	spleen	0.9*10^6^	n.d.	4	0.96	n.d.	n.d.	n.d.	n.d.

VCN, vector copy number; Lin-, lineage negative cells; SP, spleen; BM, bone marrow; THY, thymus; n.d., not done.

Accordingly, GT mice that did not overcome the untreated *oc/oc* lifespan showed low body weight (**Figure 2D**), poor bone reconstitution (**Supplementary Figures 8A–C**), absence of white and red pulp demarcation and very low transgene expression in the spleen (**Supplementary Figures 8D–F**).

These results indicate that a minimal threshold of gene correction is needed to allow long-term survival and remodeling of bone structure, in order to establish BM niches for the homing of corrected HSPCs.

### HSPC mobilization in the *oc/oc* mouse model

Another critical factor for successful application of gene therapy is the amount of HSPCs available for *ex vivo* manipulation. Anecdotal use of successful G-CSF mobilization has been reported in ARO patients (with intermediate or neuronopathic forms) or osteoclast-poor murine models (17, 18, 28). We investigated the efficacy of plerixafor (also known as AMD3100) in the *oc/oc* neonates, which present with a pathological BM niche soon after birth similarly to *TCIRG1*-defective patients. Plerixafor is a selective antagonist of stromal cell derived factor-1 (SDF-1, also known as C-X-C motif chemokine 12, CXCL12), involved in HSPC retention in BM, that is largely used in HSCT and GT settings to induce the HSPC mobilization (29, 30). We treated *oc/oc* and WT pups by subcutaneous injection of 5 mg/kg plerixafor and euthanized them one hour after the injection, when the mobilization peak is expected in adult mice (31). Mice were treated at postnatal day (PND) 3 or 7, to mimic two different stages of disease progression. At termination, we collected whole blood to perform white blood cell (WBC) counts, CFU assay and immunophenotype. At steady state, WBC counts/µl of blood were similar in WT and *oc/oc* mice. After plerixafor mobilization, we observed WBC increase in PND 7 WT mice, while a non-statistically significant trend to increase was observed on PND 3 in WT pups. On the other hand, mobilization did not affect total WBC counts in most *oc/oc* mice (**Figure 6A**). We performed CFU assay from peripheral blood to assess the number of circulating HSPCs. A statistically significant increase of CFU/µl blood was observed at PNDs 3 and 7 in WT mice (**Figure 6B**), whereas in *oc/oc* the increase in CFU counts was observed only at PND 7 (**Figure 6C**).

**Figure 6:**
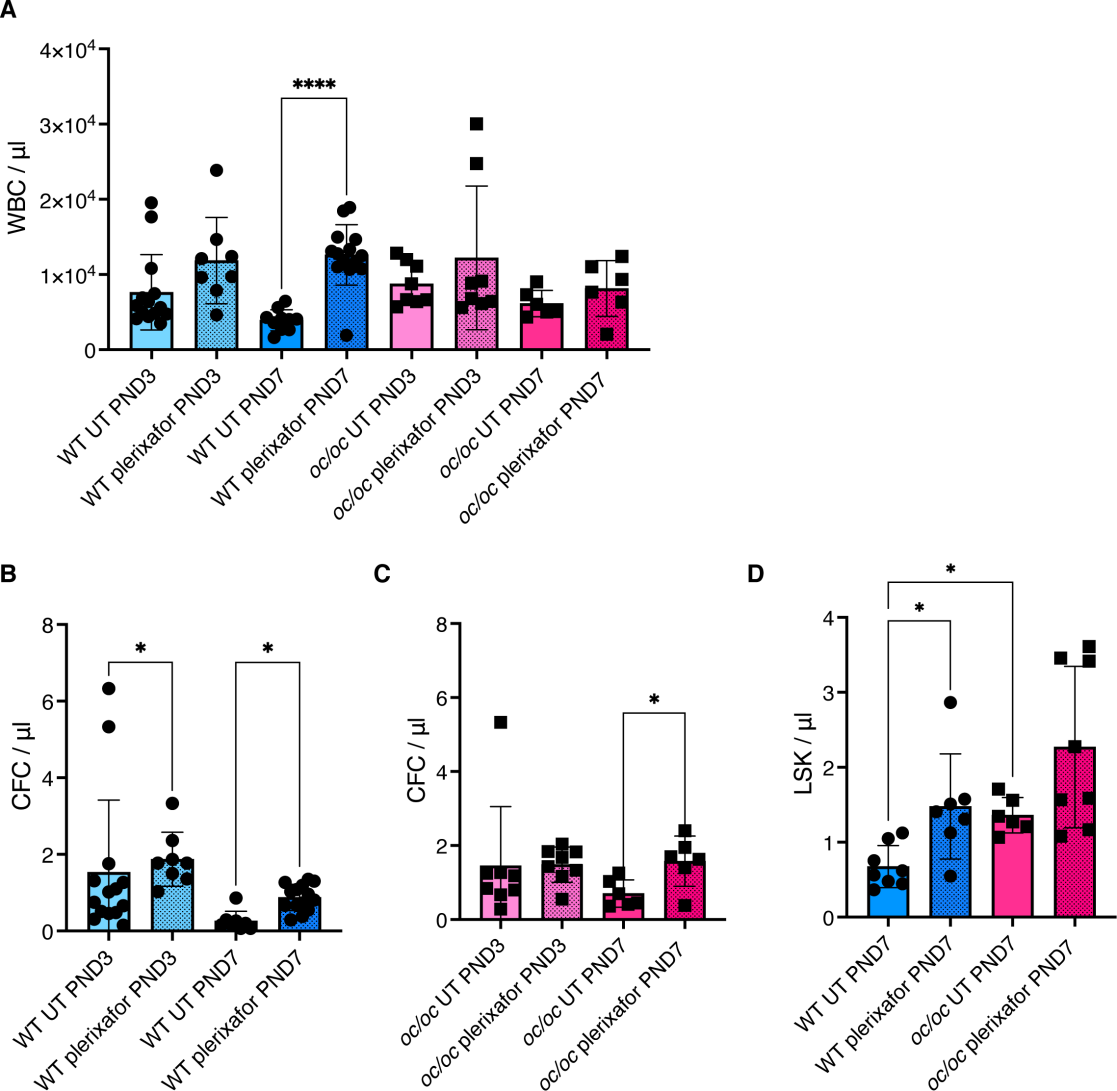
Plerixafor mobilization of hematopoietic stem and progenitor cells (HSPCs). **(A)** White blood cell (WBC) count per μl of blood of WT and *oc/oc* pups untreated (UT) or subcutaneously injected with a dose of 5 mg/kg of plerixafor (AMD3100) at post-natal day (PND) 3 and 7. **(B)** Counts of colony forming units (CFUs) for each μl of whole blood of WT mice at PND 3 and 7. **(C)** Counts of colony forming units (CFUs) for each μl of whole blood of *oc/oc* mice at PND 3 and 7. **(D)** Absolute counts of Lin- Sca1+ cKit+ (LSK) HSPCs per μl of blood at PND 3 and 7. Bars indicate mean ± SD. Statistical significance was determined by non-parametric one-way ANOVA with Dunn’s multiple comparison post-test. *p<0.05, ****p<0.0001.

To assess the composition of peripheral blood after mobilization, we performed flow cytometry analysis at PND 7, due to the technical limitation of low volumes of blood available from PND 3 pups. We observed a statistically significant increase of primitive Lin- Sca1+ cKit+ (LSK) counts/µl in plerixafor-treated WT mice, and a trend to increase in mobilized *oc/oc* mice (**Figure 6D**), in line with the results of the CFU assay. Importantly, untreated *oc/oc* pups have an LSK absolute count higher that WT steady-state littermates and comparable to the mobilized WT ones, suggesting a spontaneous circulation of HSPCs in the presence of narrow fibrotic BM niche. This observation is consistent with the high frequency of CD34+ cells found in the peripheral blood of ARO patients, which is comparable to that observed in healthy individuals after drug-induced mobilization (13, 17, 18).

These data suggest that plerixafor-based mobilization in osteopetrosis could further increase the egress of HSPCs from their hematopoietic niches, favoring the collection of adequate numbers of HSPCs for *ex vivo* manipulation.

### Efficacy of non-genotoxic conditioning

Before GT or allogeneic HSCT, pre-transplant conditioning is required for myeloablation but needs to be carefully optimized to reduce the risk of toxicity in these severely affected patients. We tested an antibody-drug conjugate formed by the saporin toxin and the anti-CD45 antibody (CD45-SAP), that had been successfully tested in adult WT and disease mouse models (23, 32–37).

WT newborns (PND 0) were treated with CD45-SAP conjugate and HSPC depletion was evaluated in hematopoietic organs after 2 days, at the time corresponding to transplant. Control group received total body irradiation (IRR) on PND 2, and depletion was evaluated after 2 hours (**Supplementary Figure 9A**). Due to the limited number of cells available at PND 2, we used the CFU assay to estimate the number of residual HSPCs after conditioning. BM showed statistically significant reduced CFU output after CD45-SAP treatment, as well as after IRR (**Figure 7A**). Reduced counts of CFUs were also observed in the peripheral blood and spleen (**Figures 7B, C**). Histological evaluation showed no lesions and basal apoptosis levels in BM, brain and kidney after non-genotoxic conditioning, unlike with irradiated samples (**Supplementary Figures 9B–E**).

**Figure 7:**
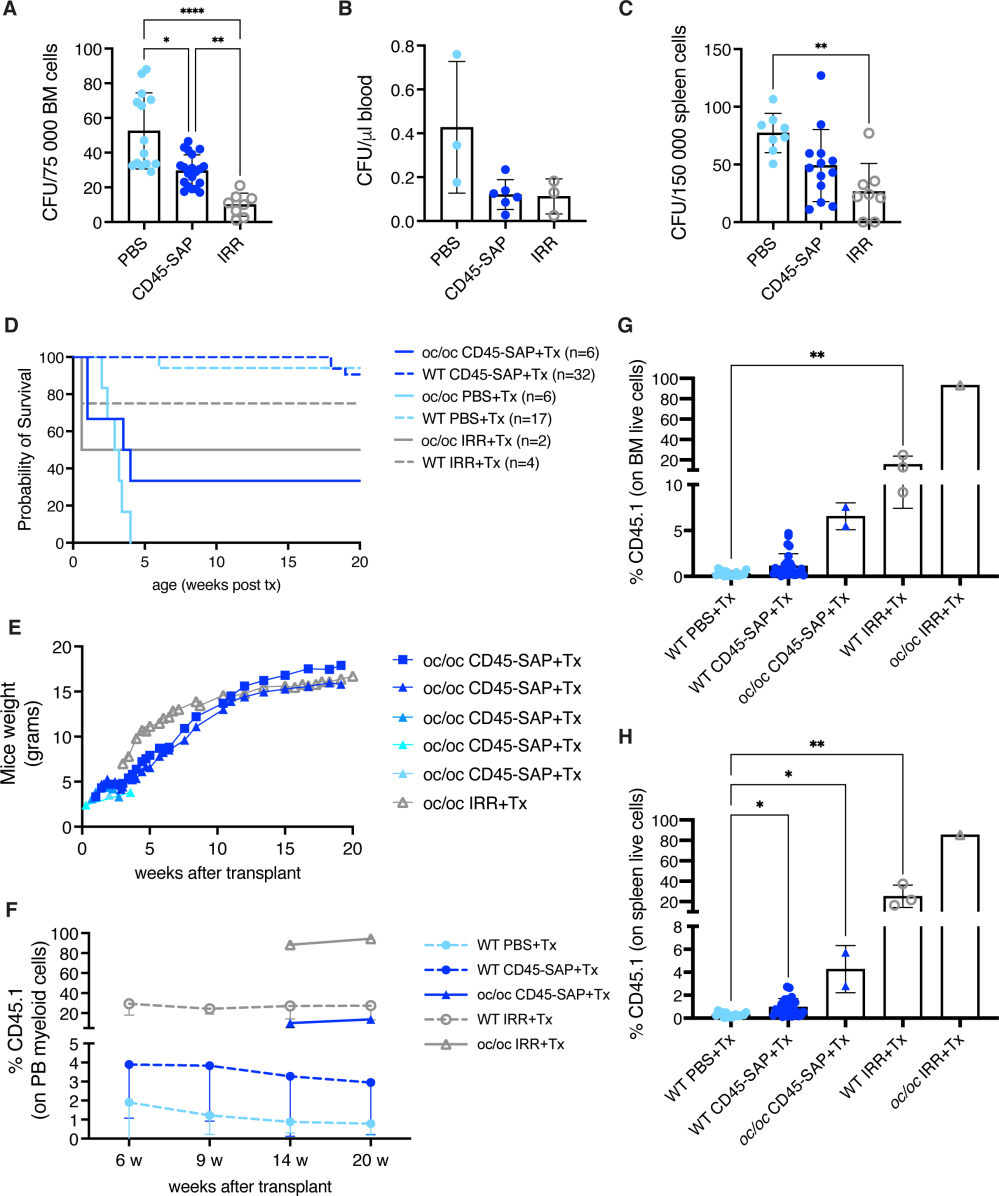
Non-genotoxic conditioning. **(A)** Counts of colony forming units (CFUs) obtained from 0.75*10^5^ bone marrow (BM) cells of the indicated groups at day 2 (before transplant). **(B)** CFU counts per μl of blood at day 2. **(C)** CFU counts obtained from 1.5*10^5^ spleen cells at day 2. **(D)** The Kaplan-Meier curve shows the survival of WT (dashed lines) and *oc/oc* (continuous lines) mice unconditioned (PBS-injected), irradiated (IRR) or CD45-saporin treated and transplanted with total BM cells. The number of mice of each group is indicated. **(E)** Weight of the *oc/oc* mice after transplant. **(F)** Mean frequency of donor-derived CD45.1 cells in the peripheral blood of transplanted mice overtime. **(G)** Frequency of donor derived CD45.1 cells in the BM of transplanted mice. **(H)** Frequency of donor derived CD45.1 cells in the spleen of transplanted mice. **(A, C)** and **(G, H)**: bars indicate mean ± SD. Panel **(F)**: each dot represents the mean ± SD. Statistical analysis A, C, G, H: non-parametric one-way ANOVA with Dunn’s multiple comparison post-test. *p<0.05, **p<0.01, ***p<0.001, ****p<0.0001.

To evaluate if the observed depletion was sufficient to allow engraftment of donor cells in WT and *oc/oc* pups, we transplanted total BM WT cells, mismatched for the CD45 allele, after CD45-SAP conditioning or IRR (**Supplementary Figure 10A**). Unconditioned WT and *oc/oc* mice were included as controls. First, we observed the outcome of the different conditioning regimens in terms of survival. In WT pups, CD45-SAP conditioning did not have an adverse impact, similarly to unconditioned control group, while 25% of irradiated WT mice showed early mortality due to acute toxicity. CD45-SAP conditioning regimen allowed the long-term survival of 2 out of 6 *oc/oc* mice, similarly to pups conditioned with standard IRR (**Figure 7D**), while all the *oc/oc* pups transplanted without conditioning died before 4 weeks of age, indicating that conditioning is indeed necessary for donor cell engraftment. In *oc/oc* mice, the increase in body weight was comparable in the conditioned groups (**Figure 7E**), and in line with the results obtained after IRR and GT or WT Lin- transplant (**Figure 2D**). We monitored the donor chimerism in peripheral blood, starting from 14 weeks after transplant for *oc/oc* mice, due to the technical limitation of blood drawing from very small *oc/oc* mice. CD45-SAP conjugate allowed the engraftment of donor CD45.1 cells, albeit at lower levels than those obtained after irradiation (**Figure 7F**). Notably, engraftment levels in *oc/oc* mice were higher than those in WT recipients, suggesting an advantage for WT cells differentiating into functional osteoclasts and establishing a functional niche in the osteopetrotic bone. We detected a proportion of CD45.1 engrafting cells in hematopoietic organs (**Figures 7G, H** and **Supplementary Figure 10B**) in line with the peripheral blood. Importantly, donor hematopoietic stem cells were detected in the BM of mice conditioned with CD45-SAP immunotoxin (**Supplementary Figure 10C**). CD45-SAP conditioning did not impact on the immune subset distribution of hematopoietic organs (**Supplementary Figure 11**). Micro-CT analysis revealed bone architecture and BMD comparable between WT and *oc/oc* CD45-SAP conditioned animals at termination (**Supplementary Figure 10D**).

## Discussion

Autosomal recessive osteopetrosis is a severe disease with high risk of post-transplant complications, despite the recent advances in allogeneic transplantation procedures (9). To minimize the immunological complications, lentiviral vector gene therapy has been proposed as an alternative treatment (13, 14).

In this paper, we exploited the murine model of *Tcirg1*-defective osteopetrosis to test the efficacy of *ex vivo* gene therapy, as well as to optimize the HSPC mobilization and pre-transplant conditioning, fundamental for a successful outcome of the GT protocol. We observed that GT *oc/oc* mice overcame their expected lifespan and 50% survived long-term, comparable to the 33% of *oc/oc* mice that survived long-term after HSCT. These results are in line with those previously published with LV_EFS.TCIRG1, that allowed 75% survival after GT (15). Notably, we observed long-term survival despite the low VCN retrieved in the organs of most animals, differently from Löfvall and colleagues (15) that obtained higher VCN in the BM with their 2-hit transduction protocol. We hypothesized that the low transduction efficacy could account for the variable number of corrected HSPCs and consequently for variable bone reconstitution, as observed by histology and micro-CT. Another possible explanation is that the low number of transplanted cells limited the rate of corrected BM niches. In particular, GT4 and GT6, the two mice with the best outcome, had the higher VCN *in vivo* and received the higher cell dose, respectively. The other GT mice received 0.7-1.3*10^6^ cells and had low VCN in the hematopoietic organs (**Table 1**). They showed partial or limited improvement of bone architecture and hematological paramenters, possibly suggesting low levels of correction, both in terms of engrafted cells and transgene expression. In line with this hypothesis, variable reconstitution and survival were also observed by Richter group after transplantation of less than 2x10^6^ GT cells/mouse (38). In those experiments, survival did not correlate with the high transduction efficiency, induced by a retroviral vector in which *Tcirg1* and reporter GFP were driven by the strong viral SFFV promoter (38).

The early treatment of *oc/oc* mice showed that GT is able to allow long-term survival, restore the bone architecture and cellularity, normalize the PTH levels and B cell output and, finally, reduce the extramedullary hematopoiesis (see results obtained for GT4 and GT6 and, to a lower extent for GT7 and GT12). On the other hand, only partial or limited normalization was observed in other GT mice (in particular in GT8, GT9 and GT10), highlighting the need for infusion of fair numbers of corrected cells. Accordingly, osteopetrotic patients show high rates of engraftment failure after allogeneic HSCT, as a result of the limited number of BM niches available to the homing of HSPCs (10, 39). Thus, the collection of adequate numbers of starting cells for *ex vivo* manipulation procedures is a crucial point in this disease.

Repeated blood draws coupled to *ex vivo* HSPC expansion (13) will allow collecting adequate CD34+ cell numbers for gene modification and increasing the final dosage of the infused drug product, favoring a more rapid engraftment and immune reconstitution in ARO patients, which show high rates of engraftment failure (10, 40). The use of granulocyte colony-stimulating factor (G-CSF) as HSPC mobilization drug has been reported in literature only in very few osteopetrotic patients and in *M-CSF* (*op/op)*, *c-Fos* and *Rankl* deficient models (17, 18, 28). G-CSF may have a detrimental effect on the BM due to neutrophil activation and subsequent inflammatory response (41, 42). On the other hand, plerixafor (CXCR4 inhibitor) has been shown to mobilize HSPC with more primitive transcriptional profile and higher homing capacity (43). In ARO, plerixafor could help mobilizing HSPCs from both residual BM niches and extramedullary sites, limiting the burden due to HSPC collection in very young children. We tested this hypothesis in the *oc/oc* murine model at 2 different ages, in comparison to their WT littermates. Similarly to patients, LSK cell count and CFUs per µL of blood in PND7 steady-state *oc/oc* mice are comparable to age-matched mobilized WT mice. Nevertheless, we observed HSPC mobilization in plerixafor-treated *oc/oc* mice at PND7 and at a lower extent at PND3, supporting its use in this peculiar disease.

Another critical point for the success of GT, and of allogeneic HSCT too, is the choice of proper pre-transplant conditioning. In osteopetrosis, accurate balance between effective emptying of BM niches and limiting toxicity is particularly important, due to the severe pre-existing clinical conditions and the high risk of post-transplant complications (9, 10, 39). We tested a non-genotoxic conditioning based on an antibody-drug conjugate formed by the saporin toxin and the anti-CD45 antibody for the first time in murine pups, to the best of our knowledge (23, 33–36). In WT and *oc/oc* pups, the CD45-SAP depletion efficacy appeared limited, possibly due to different pharmacokinetic and/or biodistribution from adult organisms. Nevertheless, we observed persistent engraftment of WT donor cells (mean 6.5% in BM) and long-term survival in 33% of *oc/oc* mice. Restoration of normal bone mineral density and increase of body weight were comparable to the mice transplanted after conventional IRR. These data suggest that novel conditioning regimens with safer profile could be considered for osteopetrosis.

In conclusion, here we showed that lentiviral vector gene therapy can correct the osteopetrotic phenotype of *oc/oc* mice. Moreover, the use of mobilization drugs and the optimization of pre-transplant conditioning could increase the feasibility of the GT protocol and improve the chances of a successful outcome in this severe disease. These results will be instrumental to optimize the gene therapy for ARO patients.

## Data Availability

The original contributions presented in the study are included in the article/**Supplementary Material**. Further inquiries can be directed to the corresponding authors.

## Supplementary material

Supplementary Figure 1 Bone histomorphometric analysis of the femur. **(A)** Thickness of the cortical bone. **(B)** Porosity of the cortical bone. **(C)** Mean thickness of trabeculae, assessed using direct 3D methods. **(D)** Trabecular number: average number of trabeculae per unit length. **(E)** Trabecular separation: mean distance between trabeculae, assessed using direct 3D. Labels indicate the identification numbers of GT mice. Bars indicate mean ± SD. GT7: cortical parameter quantification not possible. GT8: scanned but not possible to quantify. GT9 and GT10: scanned but not quantified. GT12: not done.

Supplementary Figure 2 Bone marrow (BM) immune subsets. **(A)** Percentage of myeloid cells. **(B)** Absolute count of myeloid cells. **(C)** Percentage of hematopoietic stem cells (HSCs). **(D)** Absolute count of HSC. Labels indicate the identification numbers of GT mice. GT8: not done. Bars indicate mean ± SD. Statistical analysis: non-parametric one-way ANOVA with Dunn’s multiple comparison post-test. *p<0.05, **p<0.01.

Supplementary Figure 3 Histological analysis of the spleen. **(A)** Absolute counts of total splenic cells. Labels indicate the identification numbers of GT mice. Bars indicate mean ± SD. **(B)** Representative images of hematoxylin and eosin staining of spleen.

Supplementary Figure 4 Immune subsets of the spleen. **(A)** Percentage of follicular B cells within the B cell subset. **(B)** Absolute count of follicular B cells. **(C)** Percentage of marginal zone B cells within the B cell subset. **(D)** Absolute count of marginal zone B cells. **(E)** Percentage of transitional B cells within the B cell subset. **(F)** Absolute count of transitional B cells. **(G)** Percentage of T cells. **(H)** Absolute count of T cells. Labels indicate the identification numbers of GT mice. GT8: not done. Bars indicate mean ± SD. Statistical analysis: non-parametric one-way ANOVA with Dunn’s multiple comparison post-test. *p<0.05.

Supplementary Figure 5 Immune subsets of the peripheral blood at termination. **(A)** Percentage of B cells within the B cell subset. **(B)** Absolute count of B cells. **(C)** Percentage of T cells. **(D)** Absolute count of T cells. **(E)** Percentage of myeloid cells. **(F)** Absolute count of myeloid cells. **(G)** White blood cell (WBC) absolute count. Labels indicate the identification numbers of GT mice. GT8: not done. Bars indicate mean ± SD. Statistical analysis: non-parametric one-way ANOVA with Dunn’s multiple comparison post-test. *p<0.05.

Supplementary Figure 6 Immune subsets of the thymus. **(A)** Absolute count of thymocytes. Labels indicate the identification numbers of GT mice. Bars indicate mean ± SD. **(B)** Distribution of CD4- CD8- double negative (DN), CD4+ CD8+ double positive (DP), CD4+ CD8- single positive (CD4 SP) and CD4- CD8+ single positive (CD8 SP). Mean ± SD is shown. **(C)** Distribution of double negative (DN) subsets 1, DN2, DN3 and DN4. Mean ± SD is shown.

Supplementary Figure 7 Lentiviral vector sequence detection in the spleen. Representative images of RNAscope *in situ* hybridization for the detection of the lentiviral vector WPRE (woodchuck hepatitis virus post-transcriptional regulatory element) RNA sequence. Positive signal is showed in red in both bright-field and fluorescent images and highlighted by black or white arrows, respectively. GT12: not done.

Supplementary Figure 8 GT mice terminated 4 weeks post-transplant. **(A)** Micro-computed tomography of the skull. **(B)** Representative image of hematoxylin and eosin (H&E) staining of vertebral column section. **(C)** Absolute count of the spleen. **(D)** Absolute count of the spleen. **(E)** Representative image of hematoxylin and eosin staining of spleen. **(F)** Representative images of RNAscope *in situ* hybridization for the detection of the lentiviral vector WPRE (woodchuck hepatitis virus post-transcriptional regulatory element) RNA sequence. Positive signal is showed in red in both bright-field and fluorescent images and highlighted by black or white arrows, respectively.

Supplementary Figure 9 Acute toxicity of CD45-SAP conditioning. **(A)** Experimental scheme. On the day of birth, pups are injected in the temporal vein with PBS (PBS control group) or CD45 antibody conjugated to saporin toxin (CD45-SAP). Total body irradiation control group (IRR control group) is irradiated with 300 RAD at post-natal day 2. To assess HSPC depletion, mice are terminated 2 days after PBS or CD45-SAP injection or 2 hours after irradiation. Created with BioRender.com. **(B)** Representative images of cleaved caspase-3 staining on bone marrow, brain and kidney sections. Images were acquired with 400x magnification (Leica DM2500 microscope equipped with Leica DFC310 FX camera). **(C)** Semiquantitative score of cleaved caspase-3 positivity on bone marrow. **(D)** Semiquantitative score of cleaved caspase-3 positivity on brain. **(E)** Semiquantitative score of cleaved caspase-3 positivity on kidney. Bars indicate mean ± SD. Statistical analysis: non-parametric one-way ANOVA with Dunn’s multiple comparison post-test. *p<0.05, **p<0.01.

Supplementary Figure 10 Efficacy of CD45-SAP conditioning. **(A)** Experimental scheme. On the day of birth, pups are injected in the temporal vein with PBS (PBS control group) or CD45 antibody conjugated to saporin toxin (CD45-SAP). Total body irradiation control group (IRR control group) is irradiated with 300 RAD at post-natal day 2. On day 2 (2 days after PBS or CD45-SAP injection or 2 hours after irradiation), mice are transplanted with mismatched CD45.1 total bone marrow (BM) cells by intra-liver injection. Mice are then periodically bled to assess engraftment and terminated 20 weeks post-transplant. Created with BioRender.com. **(B)** Frequency of donor derived CD45.1 cells in the thymus of transplanted mice. **(C)** Frequency of donor derived CD45.1 cells in the hematopoietic stem cells of the BM. **(D)** Representative micro-CT images and bone mineral density (BMD) quantification of the lumbar 5 vertebra. Panels **(B, C)**: bars indicate mean ± SD. Statistical analysis: non-parametric one-way ANOVA with Dunn’s multiple comparison post-test. *p<0.05, **p<0.01.

Supplementary Figure 11 Immune subset distribution at termination after CD45-SAP conditioning and transplantation. **(A)** Distribution of immune subsets (B, T, myeloid and NK cells) in recipient CD45.2 or donor CD45.1 cells of the peripheral blood. **(B)** Immune subset distribution in bone marrow. **(C)** Immune subset distribution in spleen. **(D)** Distribution of CD4- CD8- double negative (DN), CD4+ CD8+ double positive (DP), CD4+ CD8- single positive (CD4 SP) and CD4- CD8+ single positive (CD8 SP). Mean ± SD is shown.
