# Supplementary figures and images for: Correction of osteopetrosis in the neonate oc/oc murine model after lentiviral vector gene therapy and non-genotoxic conditioning
Source: Front Endocrinol (Lausanne). 2024 Sep 9;15:1450349. doi: 10.3389/fendo.2024.1450349 (PMC11416974; doi:10.3389/fendo.2024.1450349)

Supplementary Figure 1

A

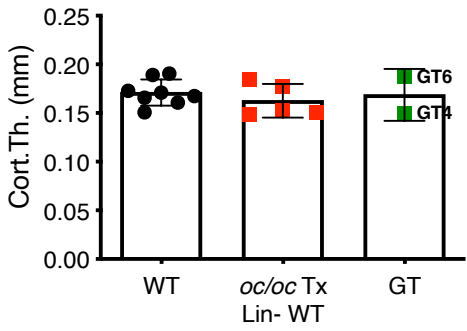

B

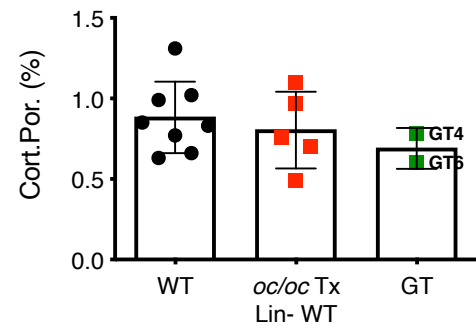

C

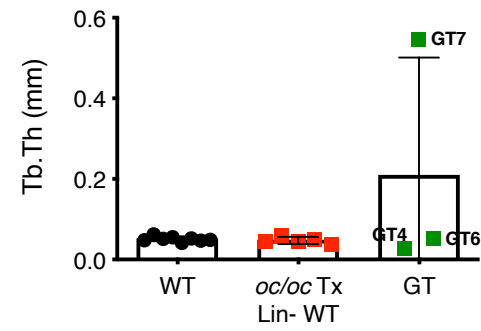

D

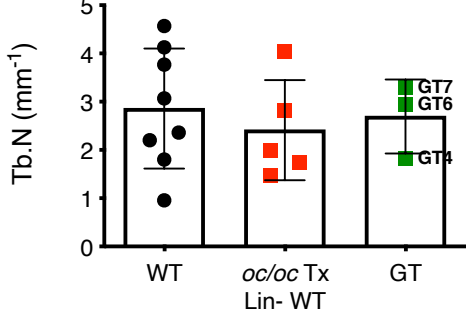

E

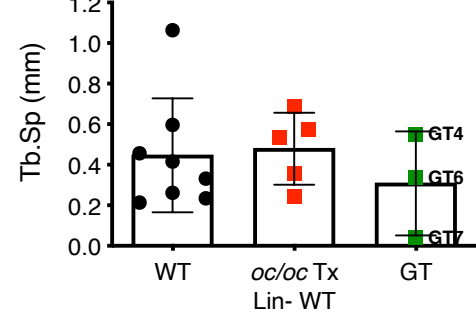

Supplement: Supplementary Figure 1 — Bone histomorphometric analysis of the femur. (A) Thickness of the cortical bone. (B) Porosity of the cortical bone. (C) Mean thickness of trabeculae, assessed using direct 3D methods. (D) Trabecular number: average number of trabeculae per unit length. (E) Trabecular separation: mean distance between trabeculae, assessed using direct 3D. Labels indicate the identification numbers of GT mice. Bars indicate mean ± SD. GT7: cortical parameter quantification not possible. GT8: scanned but not possible to quantify. GT9 and GT10: scanned but not quantified. GT12: not done. [file Image1.pdf]

Supplementary figure 2

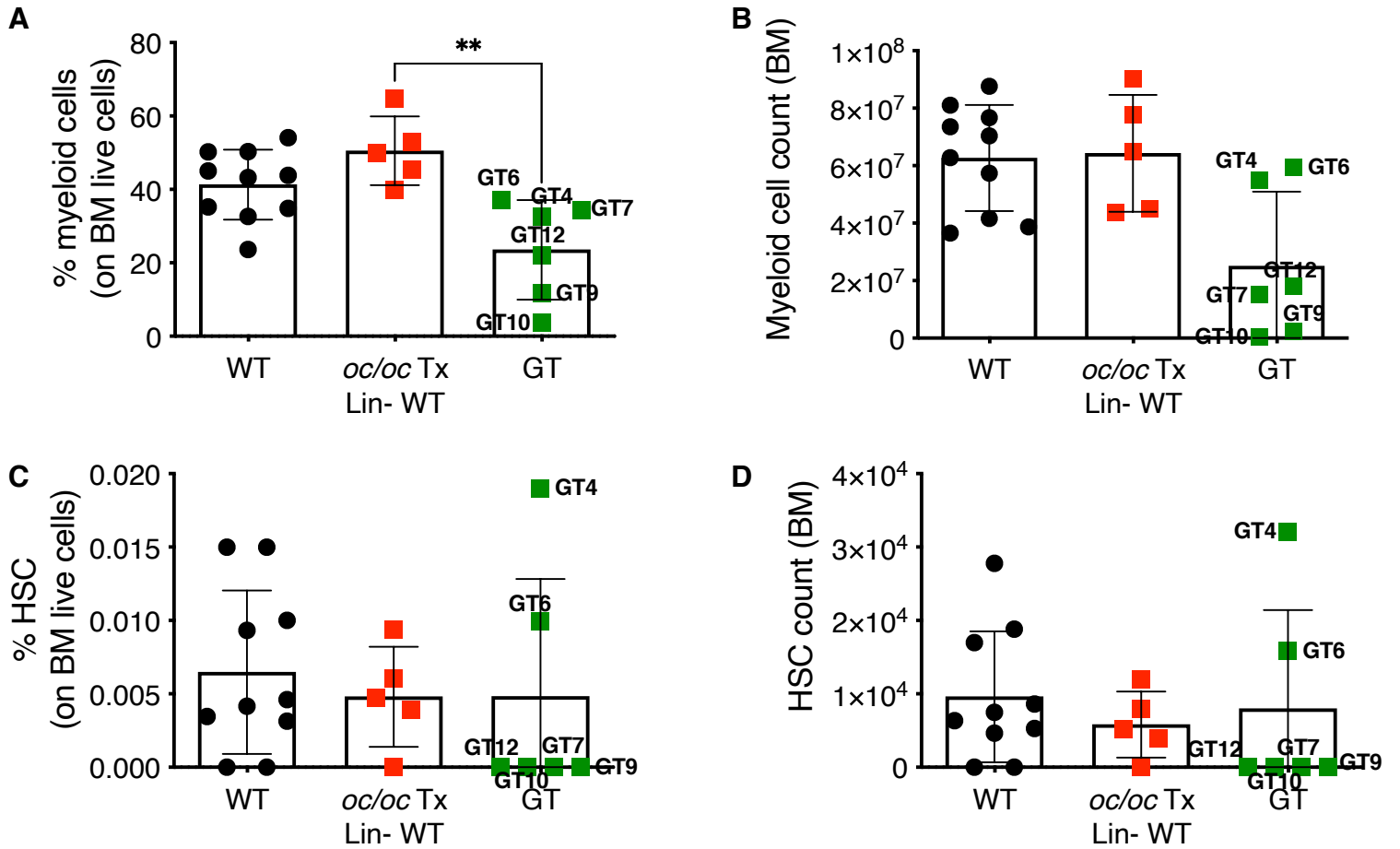

Supplement: Supplementary Figure 2 — Bone marrow (BM) immune subsets. (A) Percentage of myeloid cells. (B) Absolute count of myeloid cells. (C) Percentage of hematopoietic stem cells (HSCs). (D) Absolute count of HSC. Labels indicate the identification numbers of GT mice. GT8: not done. Bars indicate mean ± SD. Statistical analysis: non-parametric one-way ANOVA with Dunn’s multiple comparison post-test. *p<0.05, **p<0.01. [file Image2.pdf]

Supplementary figure 3

A

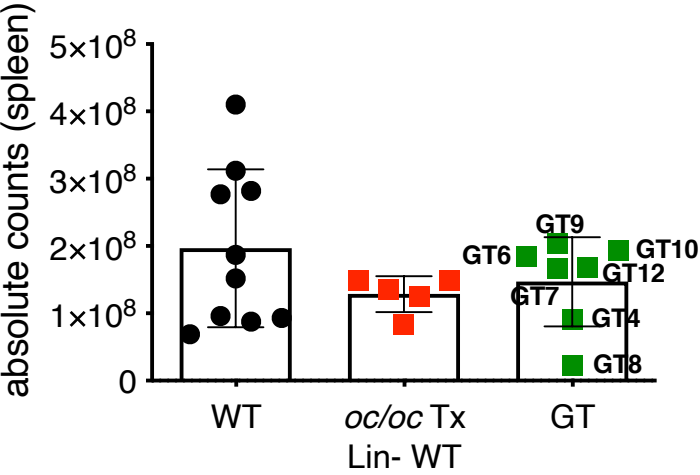

B

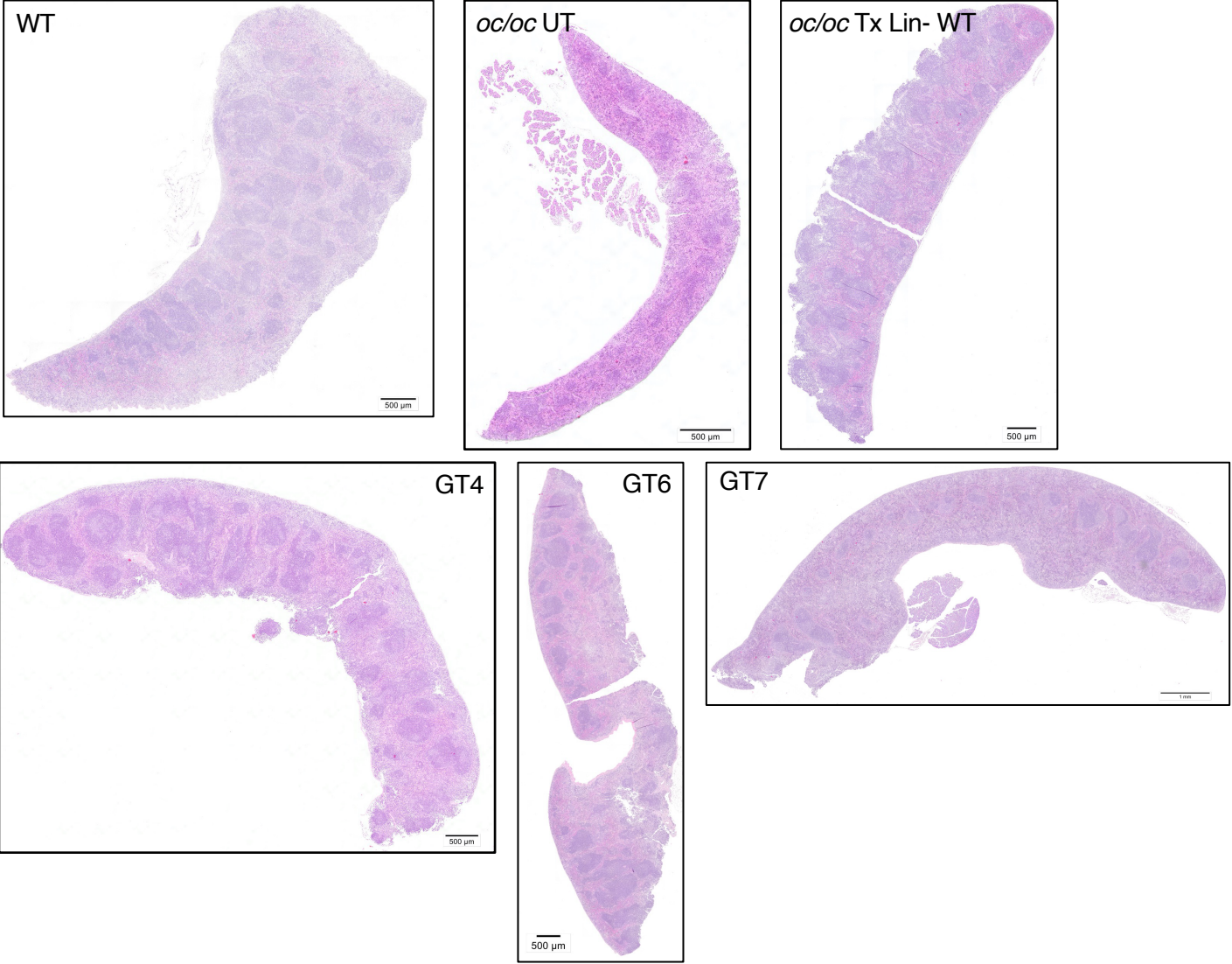

Supplement: Supplementary Figure 3 — Histological analysis of the spleen. (A) Absolute counts of total splenic cells. Labels indicate the identification numbers of GT mice. Bars indicate mean ± SD. (B) Representative images of hematoxylin and eosin staining of spleen. [file Image3.pdf]

# Supplementary figure 4

**A**

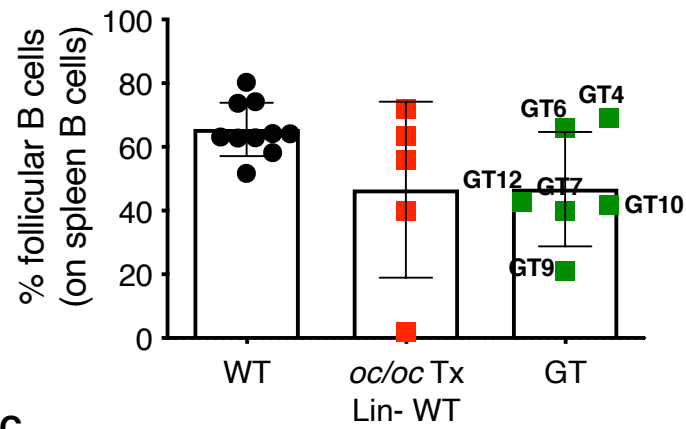

**B**

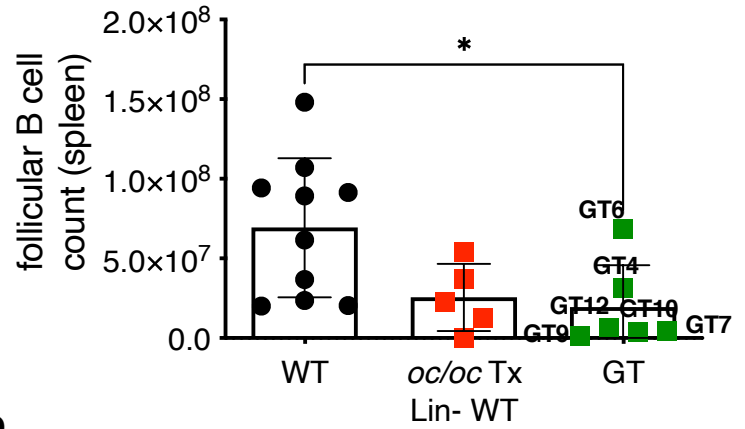

**C**

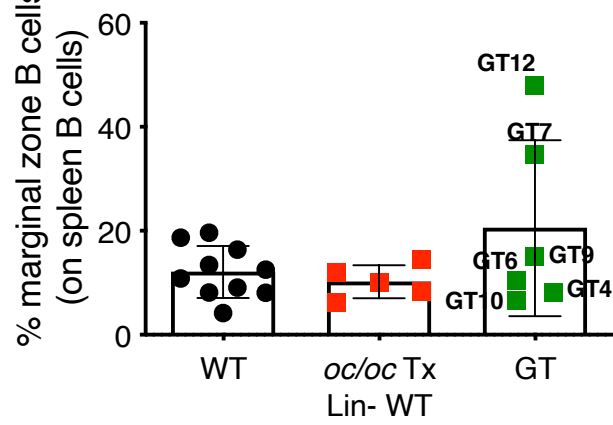

**D**

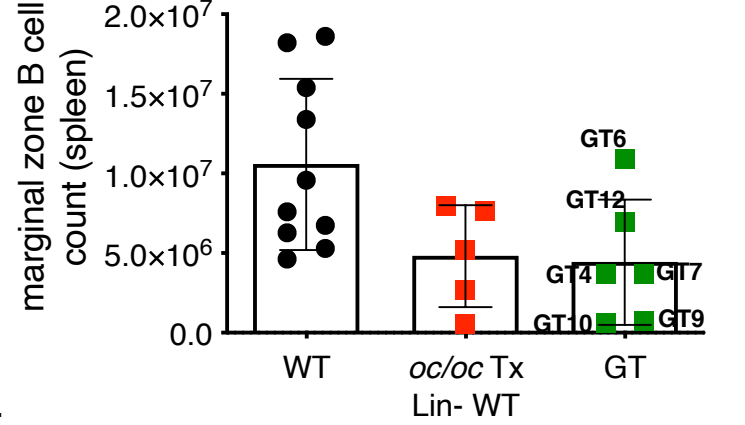

**E**

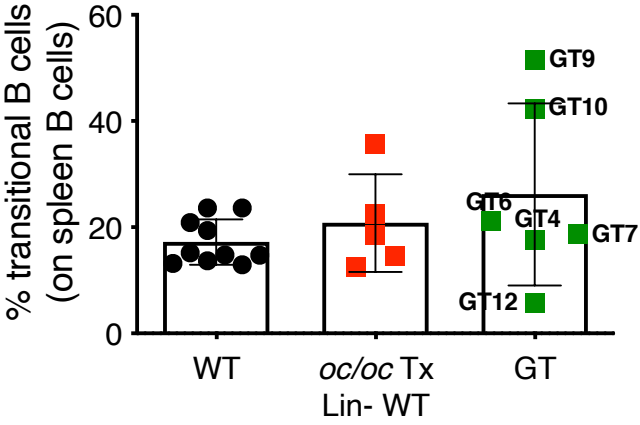

**F**

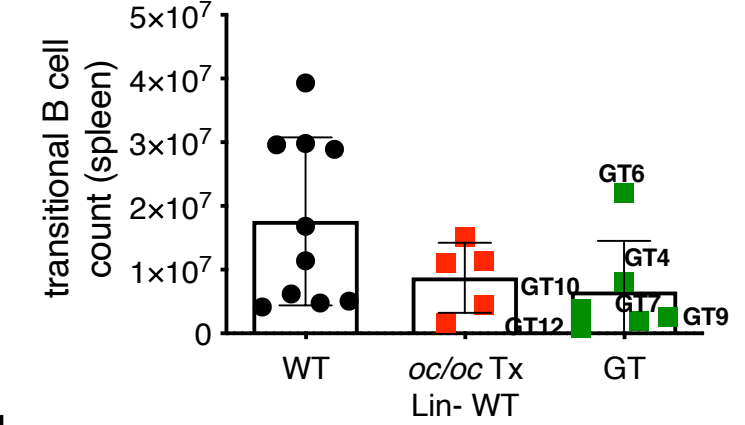

**G**

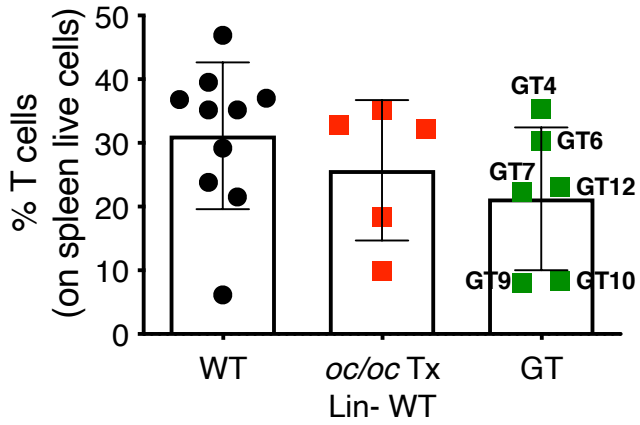

**H**

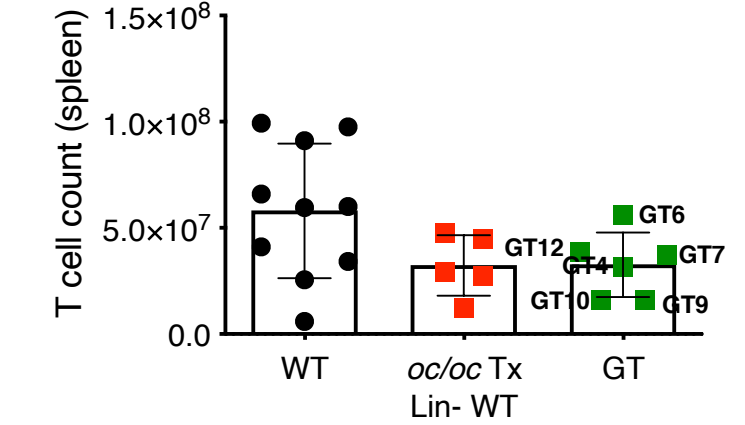

Supplement: Supplementary Figure 4 — Immune subsets of the spleen. (A) Percentage of follicular B cells within the B cell subset. (B) Absolute count of follicular B cells. (C) Percentage of marginal zone B cells within the B cell subset. (D) Absolute count of marginal zone B cells. (E) Percentage of transitional B cells within the B cell subset. (F) Absolute count of transitional B cells. (G) Percentage of T cells. (H) Absolute count of T cells. Labels indicate the identification numbers of GT mice. GT8: not done. Bars indicate mean ± SD. Statistical analysis: non-parametric one-way ANOVA with Dunn’s multiple comparison post-test. *p<0.05. [file Image4.pdf]

# Supplementary figure 5

**A**

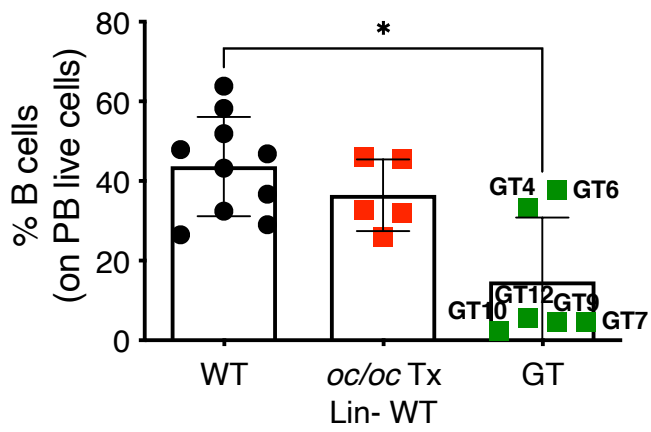

**B**

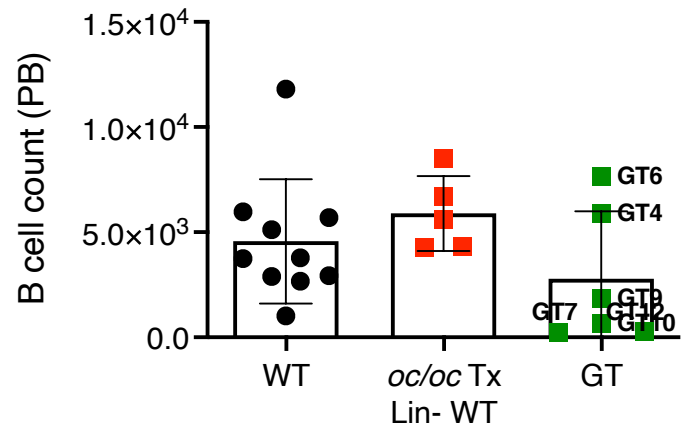

**C**

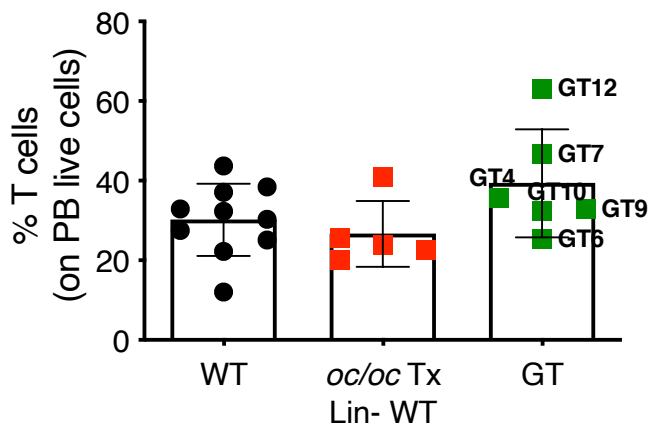

**D**

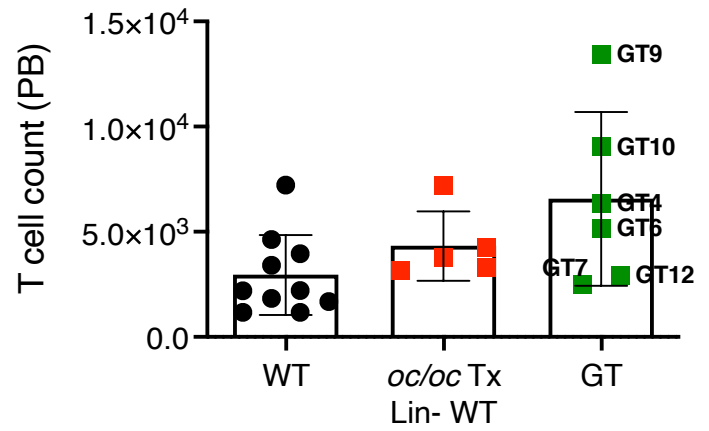

**E**

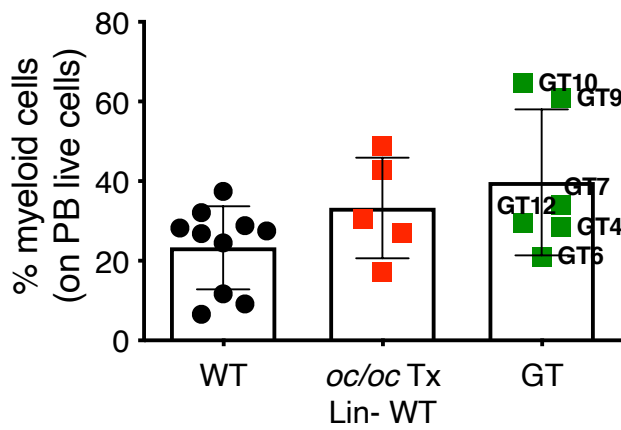

**F**

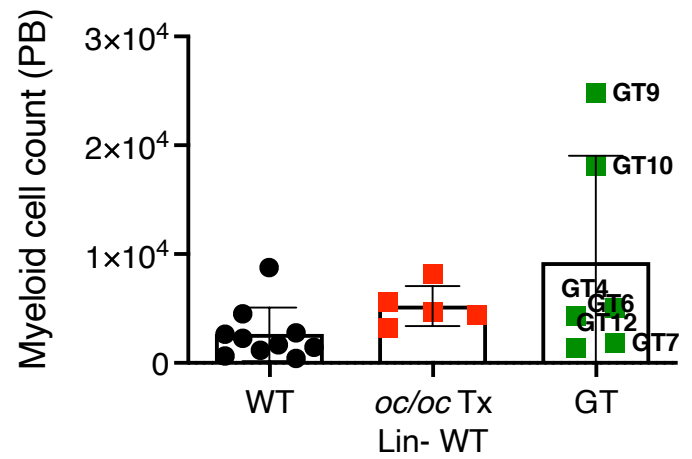

**G**

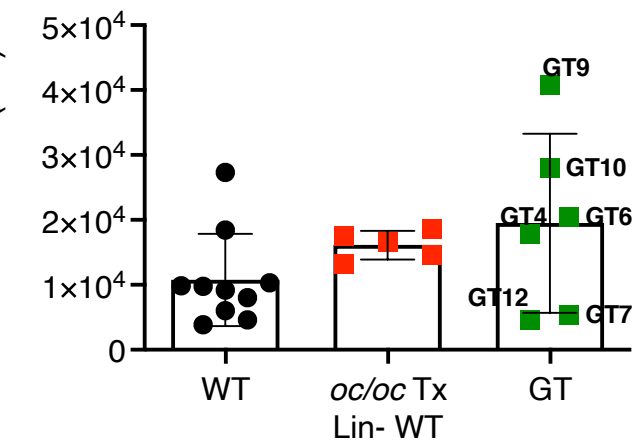

Supplement: Supplementary Figure 5 — Immune subsets of the peripheral blood at termination. (A) Percentage of B cells within the B cell subset. (B) Absolute count of B cells. (C) Percentage of T cells. (D) Absolute count of T cells. (E) Percentage of myeloid cells. (F) Absolute count of myeloid cells. (G) White blood cell (WBC) absolute count. Labels indicate the identification numbers of GT mice. GT8: not done. Bars indicate mean ± SD. Statistical analysis: non-parametric one-way ANOVA with Dunn’s multiple comparison post-test. *p<0.05. [file Image5.pdf]

Supplementary figure 6

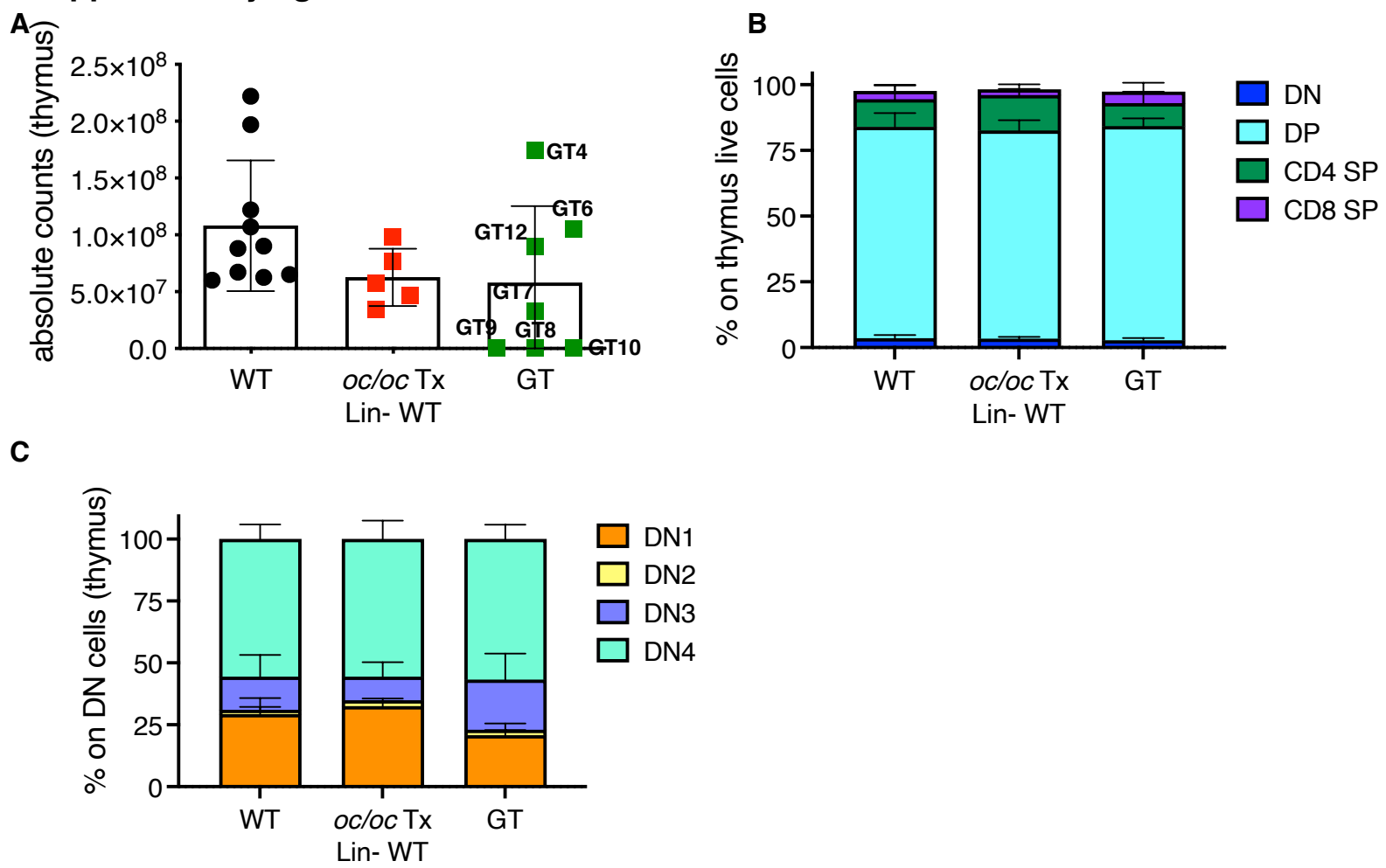

Supplement: Supplementary Figure 6 — Immune subsets of the thymus. (A) Absolute count of thymocytes. Labels indicate the identification numbers of GT mice. Bars indicate mean ± SD. (B) Distribution of CD4- CD8- double negative (DN), CD4+ CD8+ double positive (DP), CD4+ CD8- single positive (CD4 SP) and CD4- CD8+ single positive (CD8 SP). Mean ± SD is shown. (C) Distribution of double negative (DN) subsets 1, DN2, DN3 and DN4. Mean ± SD is shown. [file Image6.pdf]

Supplementary Figure 7

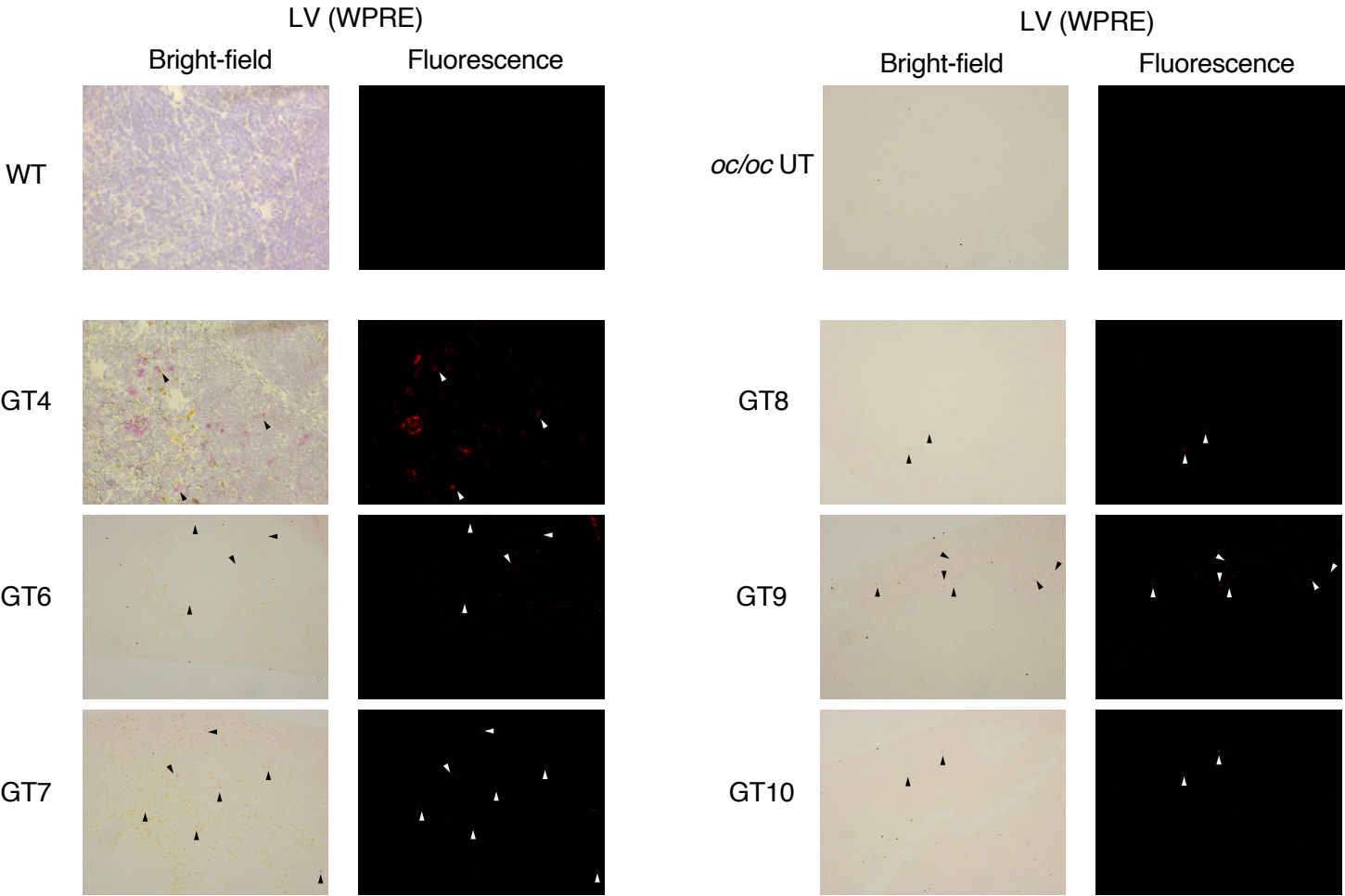

Supplement: Supplementary Figure 7 — Lentiviral vector sequence detection in the spleen. Representative images of RNAscope in situ hybridization for the detection of the lentiviral vector WPRE (woodchuck hepatitis virus post-transcriptional regulatory element) RNA sequence. Positive signal is showed in red in both bright-field and fluorescent images and highlighted by black or white arrows, respectively. GT12: not done. [file Image7.pdf]

# Supplementary figure 8

**A**

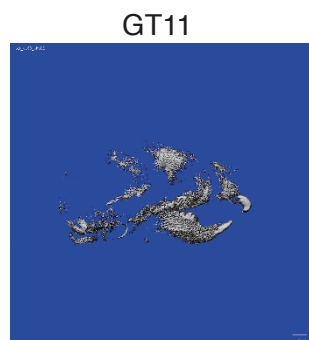

**B**

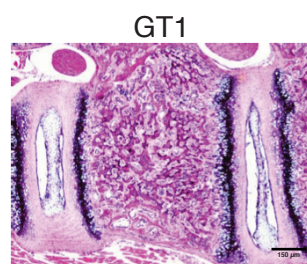

**C**

## Bone Marrow

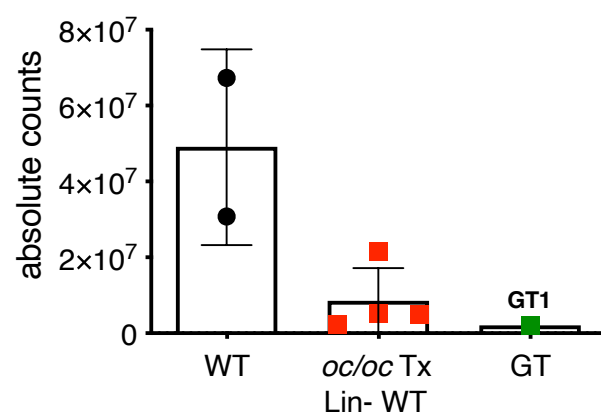

**D**

## Spleen

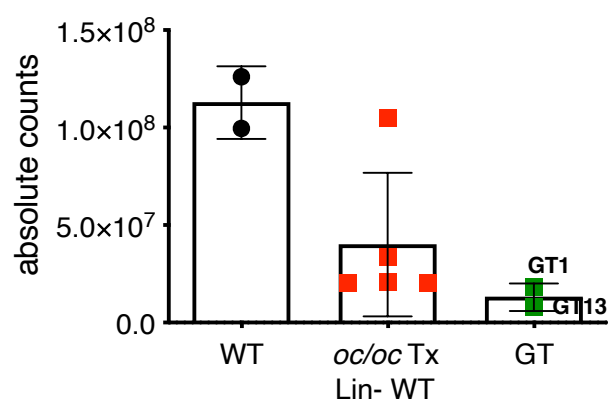

**E**

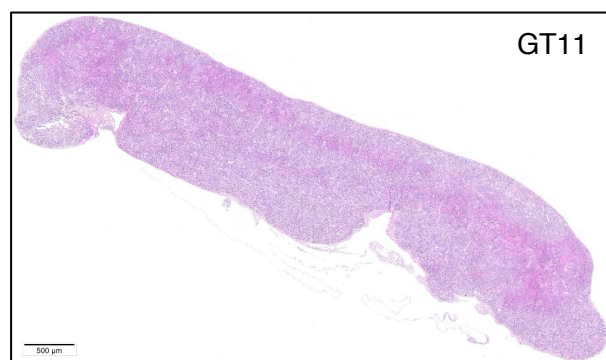

**F**

## LV (WPRE)

Bright-field

Fluorescence

GT11

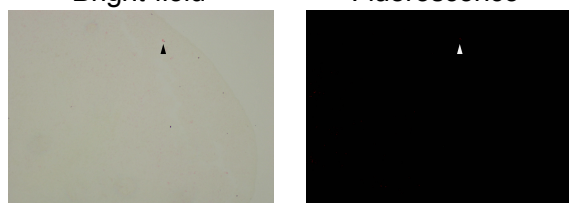

Supplement: Supplementary Figure 8 — GT mice terminated 4 weeks post-transplant. (A) Micro-computed tomography of the skull. (B) Representative image of hematoxylin and eosin (H&E) staining of vertebral column section. (C) Absolute count of the spleen. (D) Absolute count of the spleen. (E) Representative image of hematoxylin and eosin staining of spleen. (F) Representative images of RNAscope in situ hybridization for the detection of the lentiviral vector WPRE (woodchuck hepatitis virus post-transcriptional regulatory element) RNA sequence. Positive signal is showed in red in both bright-field and fluorescent images and highlighted by black or white arrows, respectively. [file Image8.pdf]

## Supplementary Figure 9

**A**

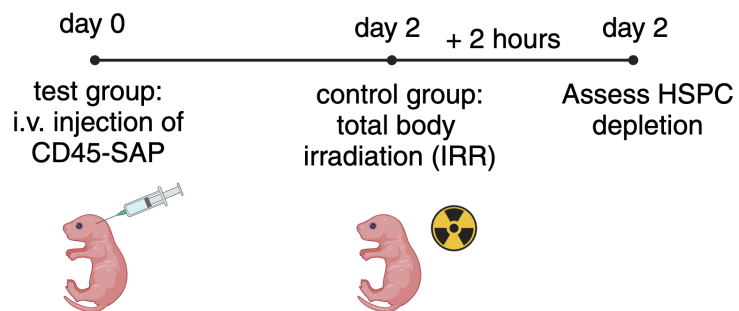

**B**

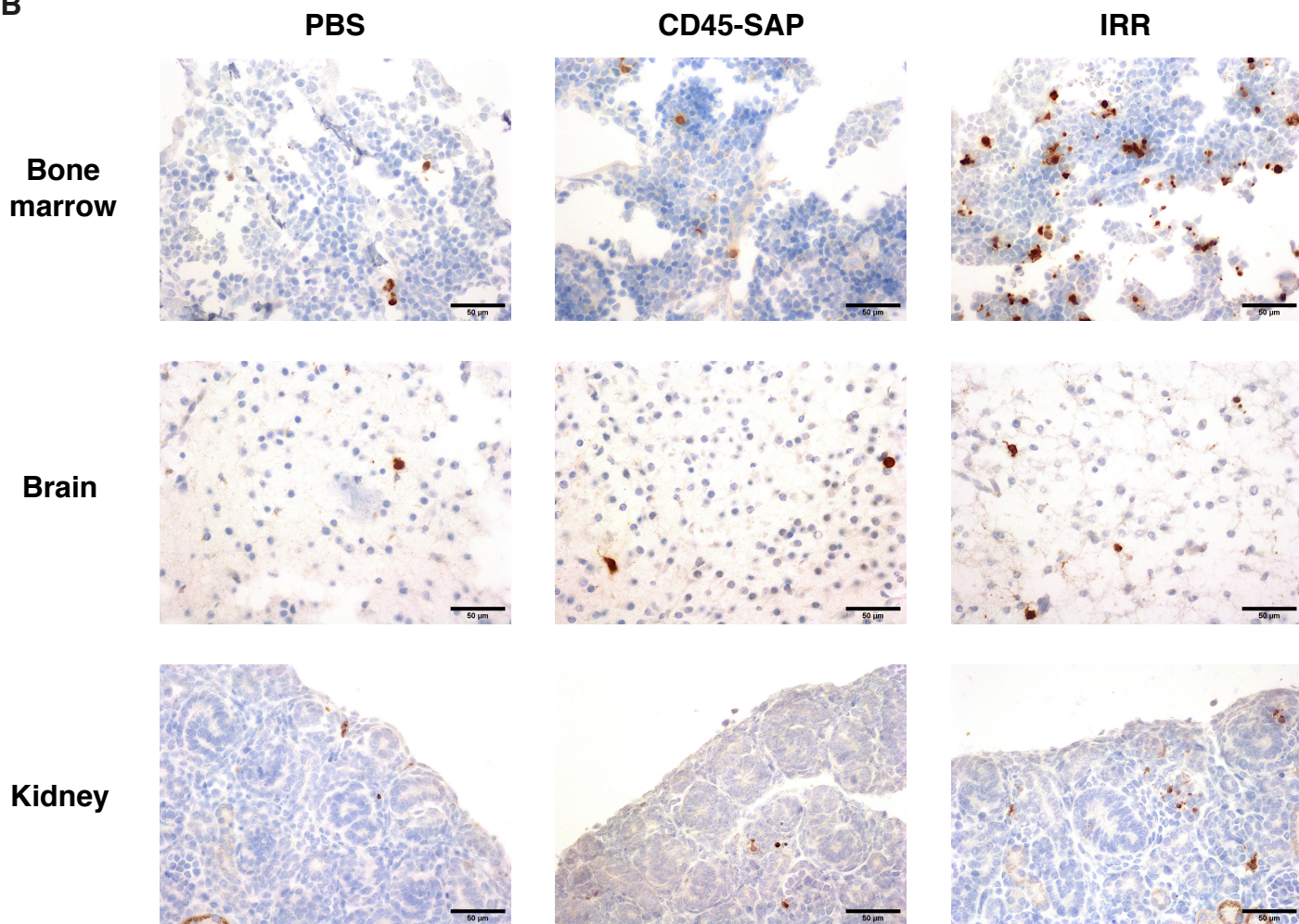

**C**

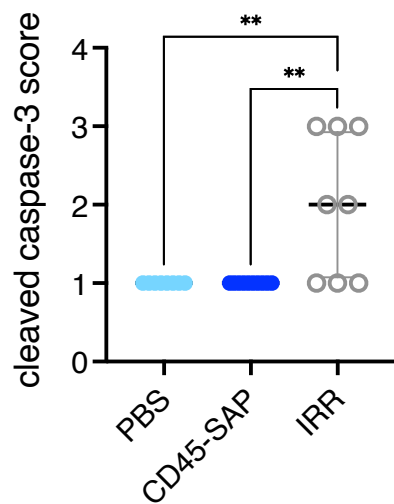

D

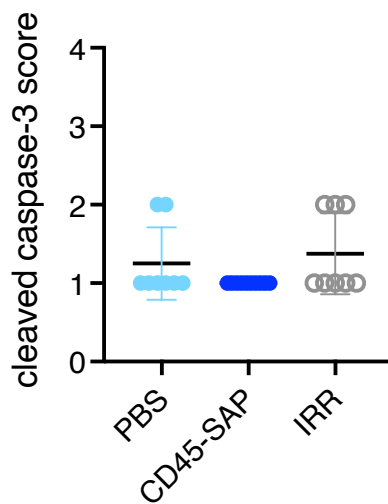

**E**

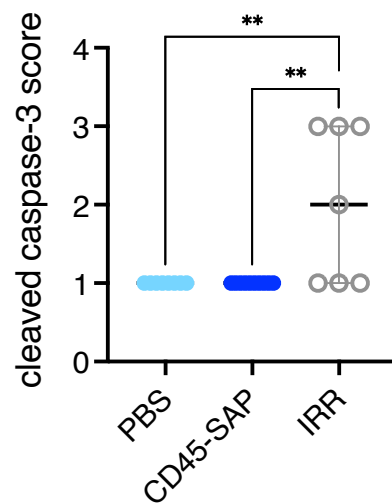

Supplement: Supplementary Figure 9 — Acute toxicity of CD45-SAP conditioning. (A) Experimental scheme. On the day of birth, pups are injected in the temporal vein with PBS (PBS control group) or CD45 antibody conjugated to saporin toxin (CD45-SAP). Total body irradiation control group (IRR control group) is irradiated with 300 RAD at post-natal day 2. To assess HSPC depletion, mice are terminated 2 days after PBS or CD45-SAP injection or 2 hours after irradiation. Created with BioRender.com. (B) Representative images of cleaved caspase-3 staining on bone marrow, brain and kidney sections. Images were acquired with 400x magnification (Leica DM2500 microscope equipped with Leica DFC310 FX camera). (C) Semiquantitative score of cleaved caspase-3 positivity on bone marrow. (D) Semiquantitative score of cleaved caspase-3 positivity on brain. (E) Semiquantitative score of cleaved caspase-3 positivity on kidney. Bars indicate mean ± SD. Statistical analysis: non-parametric one-way ANOVA with Dunn’s multiple comparison post-test. *p<0.05, **p<0.01. [file Image9.pdf]

# Supplementary figure 10

**A**

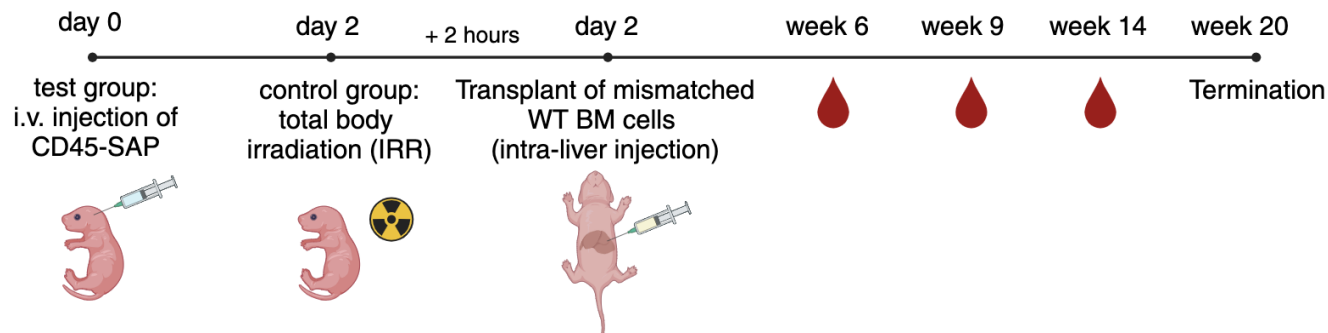

**B**

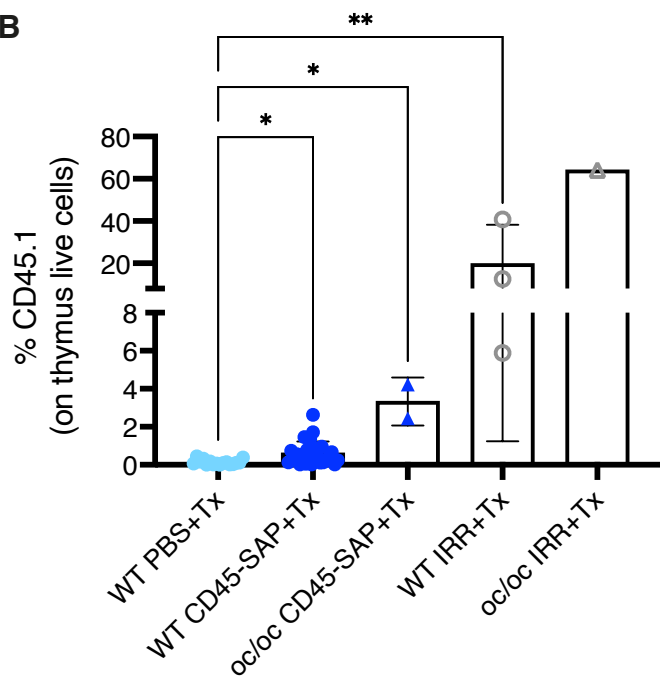

**C**

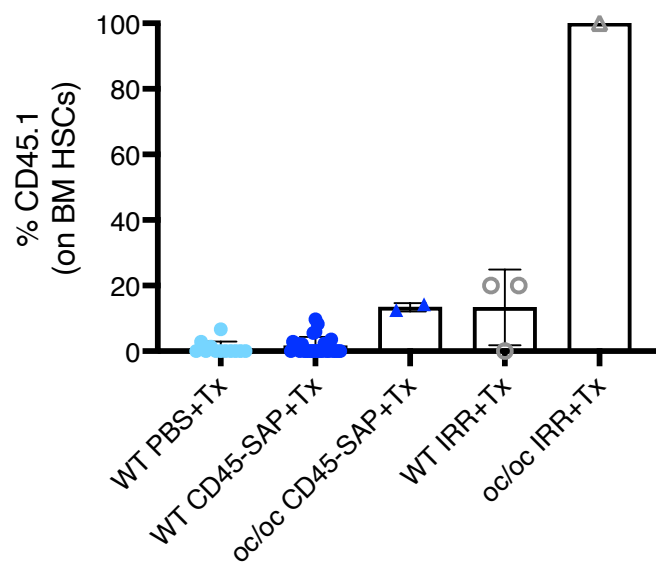

**D**

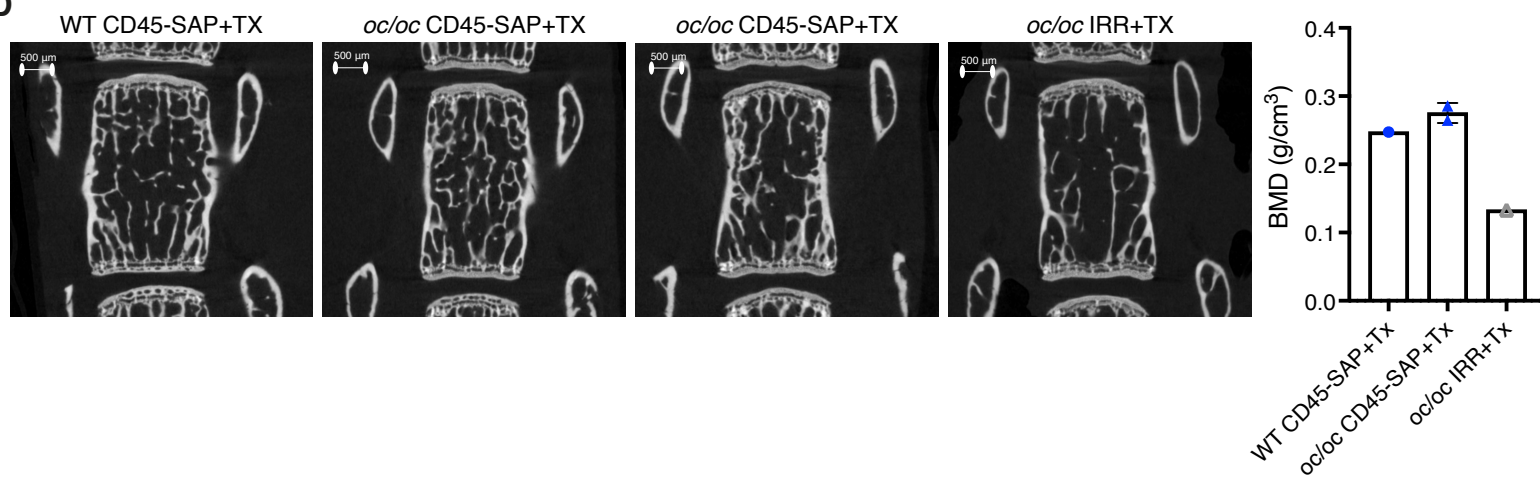

Supplement: Supplementary Figure 10 — Efficacy of CD45-SAP conditioning. (A) Experimental scheme. On the day of birth, pups are injected in the temporal vein with PBS (PBS control group) or CD45 antibody conjugated to saporin toxin (CD45-SAP). Total body irradiation control group (IRR control group) is irradiated with 300 RAD at post-natal day 2. On day 2 (2 days after PBS or CD45-SAP injection or 2 hours after irradiation), mice are transplanted with mismatched CD45.1 total bone marrow (BM) cells by intra-liver injection. Mice are then periodically bled to assess engraftment and terminated 20 weeks post-transplant. Created with BioRender.com. (B) Frequency of donor derived CD45.1 cells in the thymus of transplanted mice. (C) Frequency of donor derived CD45.1 cells in the hematopoietic stem cells of the BM. (D) Representative micro-CT images and bone mineral density (BMD) quantification of the lumbar 5 vertebra. Panels (B, C): bars indicate mean ± SD. Statistical analysis: non-parametric one-way ANOVA with Dunn’s multiple comparison post-test. *p<0.05, **p<0.01. [file Image10.pdf]

Supplementary figure 11

A

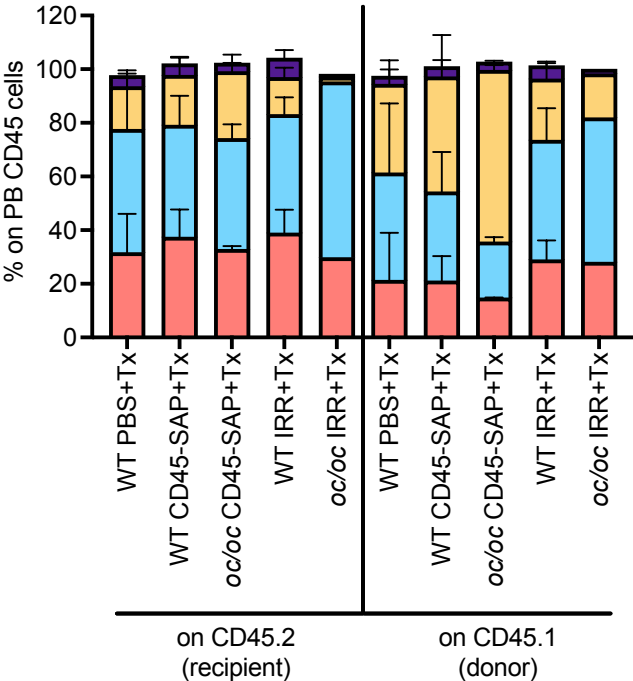

B

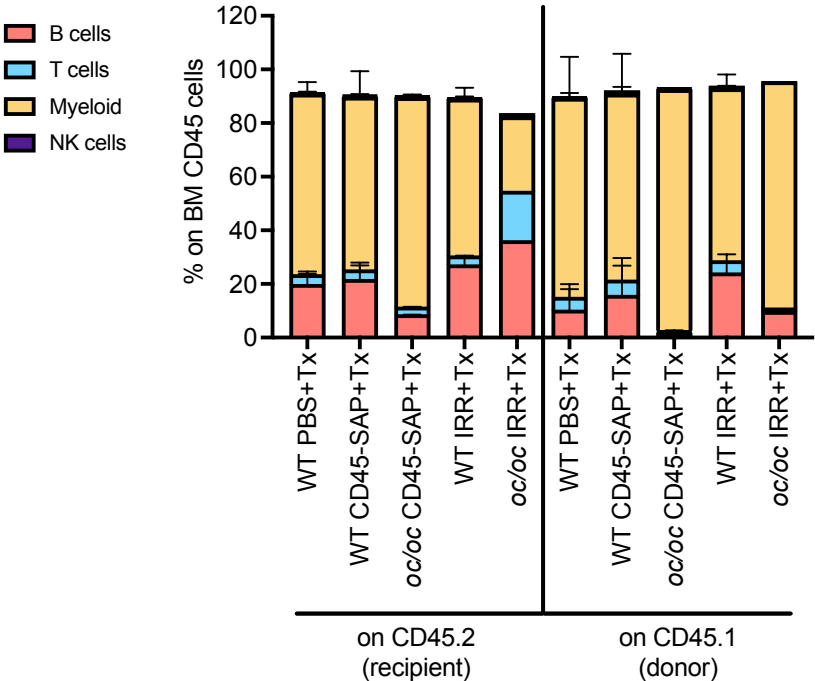

C

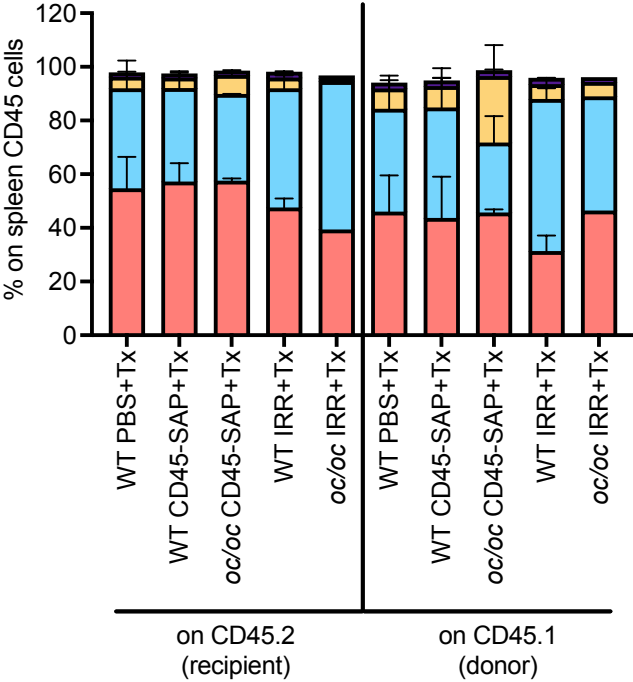

D

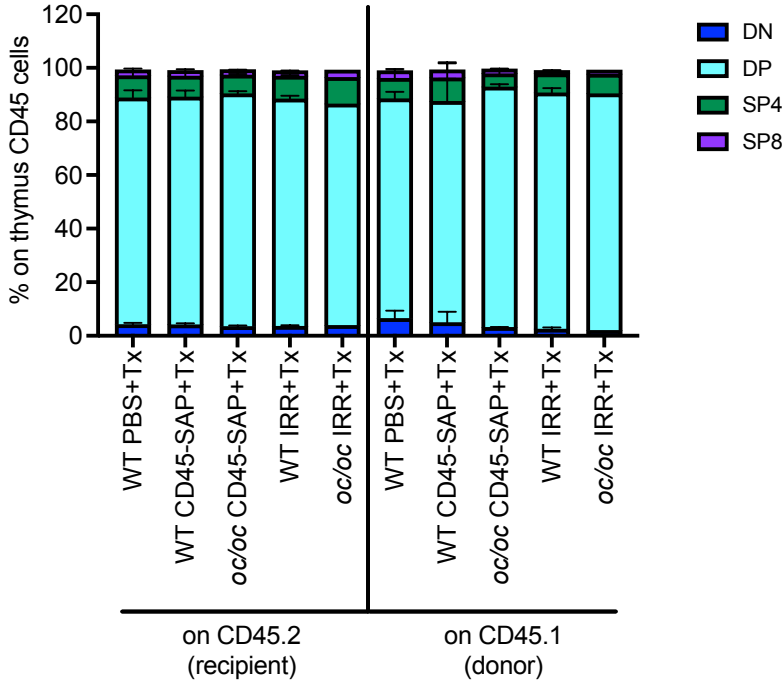

Supplement: Supplementary Figure 11 — Immune subset distribution at termination after CD45-SAP conditioning and transplantation. (A) Distribution of immune subsets (B, T, myeloid and NK cells) in recipient CD45.2 or donor CD45.1 cells of the peripheral blood. (B) Immune subset distribution in bone marrow. (C) Immune subset distribution in spleen. (D) Distribution of CD4- CD8- double negative (DN), CD4+ CD8+ double positive (DP), CD4+ CD8- single positive (CD4 SP) and CD4- CD8+ single positive (CD8 SP). Mean ± SD is shown. [file Image11.pdf]
